# Overexpression of *MdIAA9* confers high tolerance to osmotic stress in transgenic tobacco

**DOI:** 10.7717/peerj.7935

**Published:** 2019-10-31

**Authors:** Dong Huang, Qian Wang, Dingyue Duan, Qinglong Dong, Shuang Zhao, Maoxue Zhang, Guangquan Jing, Changhai Liu, Steve van Nocker, Fengwang Ma, Chao Li

**Affiliations:** 1 State Key Laboratory of Crop Stress Biology for Arid Areas/Shaanxi Key Laboratory of Apple, College of Horticulture, Northwest A & F University, Yangling, Shaanxi, China; 2 Department of Horticulture, Michigan State University, East Lansing, MI, USA

**Keywords:** Apple, Auxin, AUX/IAA gene family, Expression analysis, Abiotic stress

## Abstract

Auxin is a plant hormone that takes part in a series of developmental and physiological processes. There are three major gene families that play a role in the early response of auxin and auxin/indole-3-acetic acid (Aux/IAA) is one of these. Although the genomic organization and function of *Aux/IAA* genes have been recognized in reference plants there have only been a few focused studies conducted with non-model crop plants, especially in the woody perennial species. We conducted a genomic census and expression analysis of *Aux/IAA* genes in the cultivated apple (*Malus* × *domestica* Borkh.). The *Aux/IAA* gene family of the apple genome was identified and analyzed in this study. Phylogenetic analysis showed that *MdIAAs* could be categorized into nine subfamilies and that these MdIAA proteins contained four whole or partially conserved domains of the *MdIAA* family. The spatio-specific expression profiles showed that most of the *MdIAAs* were preferentially expressed in specific tissues. Some of these genes were significantly induced by treatments with one or more abiotic stresses. The overexpression of *MdIAA9* in tobacco (*Nicotiana tabacum* L.) plants significantly increased their tolerance to osmotic stresses. Our cumulative data supports the interactions between abiotic stresses and plant hormones and provides a theoretical basis for the mechanism of *Aux/IAA* and drought resistance in apples.

## Introduction

Auxins participate in diverse processes in the development and physiology of plants, including their cell division and differentiation, organogenesis, plant geotropic and phototropic responses, embryogenesis, apical dominance, root development, lateral root formation, axillary bud and lateral branch formation, leaf morphology, and vascular differentiation (Woodward & Bartel, 2005; Ljung, 2013). Ultimately, these processes are governed by the expression of an assortment of auxin-responsive genes (De Smet & Jürgens, 2007). The most prevalent and predominantly studied auxin, indole-3-acetic acid (IAA), enhances the transcription of several classes of early genes, which includes small auxin-up RNA (*SAUR*), Gretchen Hagen3 (*GH3*), and auxin/indole-3-acetic acid (*Aux/IAA*) gene family members (Abel & Theologis, 1996). In the absence of auxin, Aux/IAA proteins act as transcriptional repressors at promoter sites of the auxin-responsive genes, at least in part by interfering with the activity of the auxin-response factor (ARF) transcriptional regulators. Once the auxin levels rise, the AUX/IAA proteins are degraded by poly ubiquitination and, subsequently, 26S proteasome, thus depressing ARF-mediated transcription (Tiwari et al., 2001).

Most Aux/IAA proteins have four conserved interaction domains (I–IV) (Hagen & Guilfoyle, 2002). Domain I contains an ERF-associated amphiphilic repression module that interacts with the TOPLESS (TPL) corepressor (Tiwari, Hagen & Guilfoyle, 2004; Oh et al., 2014). Domain II is associated with auxin transport inhibitor response 1; mutations occurring in this domain affect their interaction and results in a low auxin response (Gray et al., 2001). Domains III and IV are able to regulate hetero-dimerization with the other Aux/IAA family members, and interactions with ARF proteins (Ulmasov et al., 1997; Shen et al., 2010).

In 1982, the first *Aux/IAA* gene was identified in the soybean (*Glycine max*) (Walker & Key, 1982) and many more members of the *Aux/IAA* genes were subsequently reported by genome-wide analyses in *Arabidopsis* (*Arabidopsis thaliana*, 34 genes), maize (*Zea mays* L., 34 genes), rice (*Oryza sativa* L., 31 genes), cucumber (*Cucumis sativus* L., 27 genes), tomato (*Solanum lycopersicon*, 26 genes), Chinese hickory (*Carya cathayensis* Sarg., 22 genes), papaya (*Carica papaya* L., 18 genes) and *Medicago* (*Medicago truncatula*, 17 genes) (Liscum & Reed, 2002; Wu et al., 2012, 2014; Yuan et al., 2018; Wang et al., 2010b; Shen et al., 2014; Jain et al., 2006; Liu et al., 2017). In *Arabidopsis*, the function of several *Aux/IAA* genes have been characterized by their corresponding mutants. *IAA7*, *IAA17*, *IAA19*, and *IAA28* are involved in determining the numbers of lateral roots, whereas *IAA14* is necessary for the formation of the lateral roots (Fukaki et al., 2002). In tomatoes, *SlIAA9* has been shown to play multiple roles in the development of the leaf shape and fruit (Wang et al., 2005), while *SlIAA15* assists in the formation of the axillary buds and the epidermis (Deng et al., 2012). In potatoes (*Solanum tuberosum* L.), the down regulation of *StIAA2* results in a greater plant height and petiole hyponasty (Kloosterman, Visser & Bachem, 2006). In rice, *OsIAA6* is highly induced by drought stress and its overexpression (OE) in transgenic rice improves drought tolerance (Jung et al., 2015).

Apples (*Malus* × *domestica* Borkh.) are a fruit crop that is cultivated throughout the world, occupying an important economic position in the world’s fruit production (Dong et al., 2018a). Apple breeding focuses on improving the stress resistance of this crop as biological and abiotic stresses are key factors in the distribution and yield of apple trees (Mao et al., 2017). However, the *GH3* gene family is currently the only one to have been investigated in the apple (Yuan et al., 2013). Previous studies have shown that specific *Aux/IAA* genes are involved in fruit development, maturation, and drought stress responses (Liu et al., 2017). However, *MdIAAs* have not been well studied under the background of abiotic stress response. Here, we have identified 34 *Aux/IAA* genes in the apple. Our goal was to document their chromosomal location, intron-exon structure, cis-element composition, and expression under development and stress. Additionally, we describe the corresponding protein domain architecture and phylogenetic relationships among the apple Aux/IAAs and those from other plants. This study also investigated the function of *MdIAA9* by overexpressing *MdIAA9* in tobacco. Our work provides a valuable resource for further investigating the functional mechanisms of *MdIAAs* in modulating the abiotic stress tolerance of the apple.

## Materials and Methods

### Plant materials and treatments

Tissue-specific expression was monitored in the young roots, stems, fully expanded leaves, flowers, and mature fruits from 5-year-old apple plants that had been treated with bud grafting. The apple plant was the combination of a scion (*Malus domestica* Golden Delicious) and a stock plant (*Malus hupehensis*). To vary the treatments, 1 year-old plants (Golden Delicious scions and *Malus* hupehensis rootstocks) were grown in pots (diameter: 300 mm, height: 320 mm) in a greenhouse. One seedling was grown per pot and 60 pots were used per treatment. In order to achieve drought stress, irrigation for the seedlings was discontinued when the plant height reached approximately 0.5 m. The 9th–12th leaves were collected from the base of the plants when irrigation had been withheld for 0, 2, 4, 6, 8, and 10 days. A 200 mM sodium chloride (NaCl) solution was used to irrigate plants using a salt stress treatment and the samples were collected at 0, 1, 5, 10, 15, and 20 days after irrigation. For chilling treatments, plants were put into phytotrons (24 h-constant illumination and photosynthetic photon flux of 270 μmol·m^−2^·s^−1^), the temperature was adjusted to 4 °C and the samples were collected at 0, 2, 4, 8, 12, and 24 h. For abscisic acid (ABA) and IAA treatments, plants were sprayed with 100 mM ABA or 10 µM IAA and the samples were collected at 0, 2, 4, 8, 12, and 24 h. The samples were frozen immediately in liquid nitrogen, then stored at −80 °C until RNA analysis occurred. The seedlings of the wild type (“NC89” tobacco) were used for genetic transformations. Transgenic plants and wild types were cultured in a growth chamber under a 16-h photoperiod at 23 °C. A total of 15 day old plants were grown on Murashige and Skoog (MS) agar medium supplemented with 0 or 200 mM of mannitol and left to grow for 13 day for the assay of osmotic stress. Seedling growth parameters, such as root lengths, fresh weights, and relative electrolyte leakage (REL), concentrations of chlorophyll, malondialdehyde (MDA), and proline were then measured. All of the experiments were repeated three times.

### Identification of apple *Aux/IAA* genes

Annotated Aux/IAA open reading frame (ORF) translations from *Arabidopsis* were obtained by searching the TAIR website (TAIR10; http://www.arabidopsis.org/) (Dong et al., 2018a). A total of 34 *Arabidopsis* Aux/IAA sequences were used as queries in a homology search of annotated ORF translations from the Genome Database for Rosaceae (GDR; http://www.rosaceae.org/) (Tian et al., 2015) using the Basic Local Alignment Search Algorithm (https://www.rosaceae.org/blast). In addition, we searched the annotated apple ORF translations using a Hidden Markov Model-based approach (HMMER v3.0) and the Aux/IAA domain signature PF02309 as maintained by the Pfam database (http://pfam.xfam.org/family/PF02309). The presence of Aux/IAA domains was confirmed in each candidate MdIAA sequence using the Pfam database (http://pfam.sanger.ac.uk/search) and NCBI-Conserved Domain Search (http://www.ncbi.nlm.nih.gov/Structure/cdd/wrpsb.cgi) Shao et al. (2014) (Dong et al., 2018b).

### Multiple sequence alignments, phylogenetic analysis, and exon/intron organization of MdAux/IAA proteins

Multiple sequence alignments were performed for 34 MdIAA protein sequences using DNAMAN 6.0.3.99 with default parameters (Zhou et al., 2017). Full-length protein sequences of *Arabidopsis* and rice were obtained from the NCBI protein database. The phylogenetic trees were estimated with the MEGA6 program (Dong et al., 2018b) using the neighbor-joining method (Saitou & Nei, 1987) with Poisson corrections and 1,000 replications for the bootstrap analysis. Genomic sequences and location data in apples were observed from the apple genome database (https://www.rosaceae.org/organism/Malus/x-domestica; Genome version 1.0). The structural features of these *MdIAA* genes which included exons/introns, exon numbers, and locations were obtained and shown using TBTools.

### Cloning of MdIAAs and gene expression analysis

Total RNA was extracted from frozen samples using the cetyl trimethyl ammonium bromide method (Wang et al., 2017a). Two µg of total RNA was used for synthesizing the first-strand cDNA. MdIAA ORF sequences were obtained using RT-PCR, with fully expanded leaves from the Golden Delicious apple as an RNA source, in addition to 17 gene-specific primer pairs as listed in Table 1. For quantitative real time PCR (qRT-PCR) assays, gene-specific primers were designed and synthesized by Sangon Biotech (Shanghai, China). One μg of total RNA from each sample was used to perform reverse transcription and one μL of the product was used for PCR-amplification. All reactions contained 10 µL of SYBR^®^ Premix Ex Taq™ (TaKaRa, Kusatsu, Japan), 0.4 µL of each specific primer, 8.2 µL of ddH_2_O_2_, and 1.0 µL of cDNA template, and the reactions were run by an iQ5 instrument (Bio-Rad, Hercules, CA, USA) (Dong et al., 2018a). The PCR conditions included an initial 95 °C for 3 min, then 40 cycles of 95 °C for 10 s, 58 °C for 30 s, and 72 °C for 15 s; this was followed by 72 °C for 3 min and then 81 cycles of 7 s each, increasing by an increment of 0.5 °C from 55 to 95 °C. Three biological replicates were conducted for each treatment and the values of ΔCt were calculated using the *MdMDH* gene or *NtActin* as the endogenous control (Wang et al., 2017b). The relative expression levels of MdIAA genes were obtained according to the 2^−ΔΔCt^ method (Livak & Schmittgen, 2001) and the specificity of the amplifications was examined by dissociation curve analysis.

**Table 1:** Application of primers and sequences.

Use	Primer name	Forward primer (5′-3′)	Reverse primer (5′-3′)
Complete ORF amplification	*MdIAA4*	ATGGAGAGTGGAGGTTCT	CTAGGTTGGCTTACATCT
	*MdIAA5*	ATGTCTCCACCACTATTG	CTAGTTCCGGTTCTTGGA
	*MdIAA8*	ATGTCTATATCTTTGGAG	CTAATTACTATTTTTGCAC
	*MdIAA9*	ATGTCTCCGCCACTGCTT	CTAGTTCCTGTTCCTGCA
	*MdIAA10*	ATGGCAATGTTTGCGCAG	TCAGAGCGGGCAGCAGCCT
	*MdIAA14*	ATGGAAGGCAAGGCACAT	TCATACACCACACCCCAA
	*MdIAA15*	ATGGTTAAGTTGTATAAT	TCAGATTCCAACTTTCAC
	*MdIAA16*	ATGTTACCGGAAAATAAG	TCAGTGTGTGCTTGTGCA
	*MdIAA20*	ATGGCAATGTTTGCGCAG	TCACGGCGTGCAGCAGCCT
	*MdIAA21*	ATGGGGTTTGAAGAGACA	TCAGCTCCTGTTCTTGCAT
	*MdIAA24*	ATGTCTAGGCCACTGGAGC	TTAGTTCCGGTTCTTGCAT
	*MdIAA26*	ATGACGAGCATGCTTGGA	TCAGCTTCTGCCTTTGCAT
	*MdIAA27*	ATGGCCACTGAGATGGAGG	TCAAAGTGCCATCTTATC
	*MdIAA28*	ATGGAAGACCAGCTAAAT	TCAGAAACAAGTGCTCAA
	*MdIAA30*	ATGGAAAGCAAGGCACATG	TCATACGCCACACCCCAAA
	*MdIAA31*	ATGCAAACACAGACACAAG	TCAGCTTCTGCCCTTGC
	*MdIAA33*	ATGTCACCGCCACTGCTT	CTAGTTCCTGTTCCTGCAC
qRT-PCR	*MdIAA4*	CGACACATGGTTCAGGTGGT	CCACAAGCATCCAGTCTCCA
	*MdIAA5*	TTCACGCTCTATGGACTGCATCTC	CTGACAAGTTCGAGACCGTGGAG
	*MdIAA8*	CAAGATGGGAGCAGGGTGTC	GATAGCTGCCATAGGTTTTGAGAT
	*MdIAA9*	GGAGCTCCAGGCAGAGAGAT	CAAGGAACATCGCCCACGAG
	*MdIAA10*	CCAAGGATTCCGAGCAGAAC	CTTGGAAGGTGGAGGCTGAG
	*MdIAA14*	AGGAAGAACAGCCTCCAGGTAA	AAGGTGCTCCATCCATGCTT
	*MdIAA15*	CATGCTCCAGTTGTCGGGTG	GTGACTCCGAATCAGCTGCC
	*MdIAA16*	GACGGACTGAACTACGATGAGACG	GTGCCTTAGCTGGAGCCTTATGAG
	*MdIAA20*	AGACCACTGCAATCCGACATGAAC	TGTCTTGGTAGGTGGAGGCTGAG
	*MdIAA21*	TTGGATGCTGGTTGGCGATGTG	GTCCAACAGCCTCCTTGCTCTTC
	*MdIAA24*	CAGGGGCAAAAAGGGGAT	TGGCAGAGGAACAGAAGAAACC
	*MdIAA26*	GTGGTACTTCTACTGTGGCTGAGC	ACAACTTGTGCCTTGGCTGGAG
	*MdIAA27*	TGAAGGAGACAGGATGCTTGTTGG	TGCTGCTACGAAGACGAAGTGC
	*MdIAA28*	ACGGCGACTGGATGCTTGTTG	ACAAGTGCTCAATCCTCTGGCTTC
	*MdIAA30*	GAGCATGGATGGAGCACCTTACC	ATCTCCAACCAGCATCCAATCACC
	*MdIAA31*	ACCAACACAACAACGCAACAAGAC	CGGAGGTTCAGATCACGCTCAAC
	*MdIAA33*	AAGATGGTGACTGGATGCTTGTGG	TTCCTGTTCCTGCACTTCTCCATG
	*MdMDH*	CGTGATTGGGTACTTGGAAC	TGGCAAGTGACTGGGAATGA
pBI-121	*MdIAA9*	ACGGGGGACTCTAGAGGATCCATGTCTCCGCCA	CGATCGGGGAAATTCGAGCTCCTAGTTCCTGTT
	35S	CGCACAATCCCACTATCCTT	
	*qRT-MdIAA9*	GGAGCTCCAGGCAGAGAGAT	CAAGGAACATCGCCCACGAG
	*NtActin*	TCCAGGACAAGGAGGGTAT	CATCAACAACAGGCAACCTAG

**Note:**

ORF, open reading frames.

### Prediction of cis-acting elements in promoters

The upstream regions (1,500 bp upstream of the translational start codon) of the selected MdIAAs were used to identify putative cis-acting elements (Plant CARE database; http://bioinformatics.psb.ugent.be/webtools/plantcare/html/) (Wang et al., 2017a).

### Vector construction and plant transformation

In order to isolate the full-length cDNA of *MdIAA9* used to construct the OE vectors, we conducted RT-PCR using fully expanded Golden Delicious apple leaves. The cDNA of *MdIAA9* was cloned into pBI 121 vectors and was driven by a 35S promoter known as the cauliflower mosaic virus. For tobacco transformation, wild type (“NC89” tobacco) plants were transformed using the *Agrobacterium tumefaciens* EHA105-mediated leaf dip method (Xia et al., 2012). The PCR-positive plantlets were transplanted into soil for growing in the greenhouse. Seeds were screened with 50 mg L^−1^ kanamycin after the transgenic plants were harvested individually. Homozygous transgenic T3 plants were used in osmotic stress investigations.

### Measurement of physiological indices

Relative electrolyte leakage was determined according to the method described by Tan et al. (2017). Chlorophyll concentrations and free proline were measured using the method of Dong et al. (2018a), and MDA levels were measured as described by Wei et al. (2018).

### Statistical analysis

The experimental data obtained was analyzed using SPSS 20 software (SPSS, Inc., Chicago, IL, USA) and indicated by means ± standard deviation. Data was analyzed using One-way ANOVA and Tukey’s tests at a significance level of *P* < 0.05.

## Results

### *Aux/IAA* gene family members in apple

In order to identify AUX/IAA proteins in apples, 34 *Arabidopsis* Aux/IAA proteins were used to search the apple genome database by BLASTP. In addition, we carried out a Hidden Markov Model search based on the AUX/IAA domain signature. In all, 35 candidate genes were found. In order to further confirm and identify the conserved Aux/IAA domains, all candidate proteins were analyzed using Pfam and SMART databases. One gene, *MD15G1305900*, was not analyzed further because it contained an apparently incomplete reading frame. We obtained basic information for the remaining 34 *Aux/IAA* genes, which included gene names, locus IDs, intron numbers, ORF lengths, chromosome locations, and numbers of amino acids (Table 2). The ORF lengths of MdIAAs ranged from 498 base pairs (*MdIAA19*) to 1,158 base pairs (*MdIAA34*), with an average length of 789 base pairs. The size of MdIAA proteins were found to have a range of 165–385 amino acids. The corresponding molecular mass varied from 18.21 kD (MdIAA19) to 40.61 kD (MdIAA34).

**Table 2:** Apple *AUX/IAA* genes in this study.

Gene	Locus	Gene position	Chr no.	ORF length	Amino acids	MW	PI	Number of exons
*MdIAA1*	MD02G1027600	2109632 2111147	2	744	247	27.79	8.23	5
*MdIAA2*	MD15G1191800	15104026 15107317	15	957	318	33.29	8.19	5
*MdIAA3*	MD13G1222200	21514224 21516886	13	921	306	32.22	7.34	5
*MdIAA4*	MD10G1176400	26870201 26873035	10	888	295	31.09	6.78	5
*MdIAA5*	MD01G1045800	14633447 14638572	1	1,122	374	40.26	7.19	4
*MdIAA6*	MD17G1198300	23886616 23888516	17	618	205	22.40	7.56	5
*MdIAA7*	MD05G1205900	33515071 33516331	5	528	175	19.62	5.46	3
*MdIAA8*	MD02G1057200	4631406 4634380	2	960	319	33.22	8.07	2
*MdIAA9*	MD08G1207300	26957415 26962300	8	1,092	363	39.54	6.99	5
*MdIAA10*	MD12G1241700	31336165 31337389	12	594	197	22.27	6.59	4
*MdIAA11*	MD09G1208000	19937208 19940388	9	966	321	34.61	8.31	5
*MdIAA12*	MD16G1132500	10120494 10121822	16	603	200	22.32	8.63	4
*MdIAA13*	MD08G1111200	9774753 9777030	8	888	295	32.61	8.19	5
*MdIAA14*	MD16G1206500	19099027 19102542	16	576	191	21.38	8.19	3
*MdIAA15*	MD15G1090600	6282056 6284672	15	888	295	62.51	8.54	5
*MdIAA16*	MD09G1216100	21391959 21393811	9	615	204	22.62	7.08	4
*MdIAA17*	MD13G1137000	10537566 10539193	13	582	193	21.42	8.64	4
*MdIAA18*	MD10G1312300	39684595 39685128	10	534	177	19.96	6.84	1
*MdIAA19*	MD13G1253600	27267886 27268744	13	498	165	18.21	8.92	2
*MdIAA20*	MD04G1225000	30478283 30479519	4	591	196	22.13	5.62	2
*MdIAA21*	MD10G1192900	28909337 28912808	10	774	257	28.22	8.74	5
*MdIAA22*	MD05G1188800	31653742 31656640	5	897	298	31.01	2.68	5
*MdIAA23*	MD17G1198100	23798955 23803215	17	573	190	21.42	5.92	3
*MdIAA24*	MD17G1189100	22550880 22553660	17	996	331	35.96	8.30	5
*MdIAA25*	MD08G1151300	15483655 15485406	8	732	243	27.54	7.16	4
*MdIAA26*	MD16G1206700	19206017 19208495	16	735	244	26.75	7.88	5
*MdIAA27*	MD09G1202300	18823904 18826992	9	930	309	34.21	8.72	5
*MdIAA28*	MD10G1193000	28924800 28926049	10	522	173	19.29	7.80	3
*MdIAA29*	MD17G1183500	21576812 21580307	17	975	324	35.79	8.56	5
*MdIAA30*	MD13G1204700	18453283 18454792	13	573	190	21.34	8.86	3
*MdIAA31*	MD13G1205000	18556276 18559279	13	963	320	35.28	9.11	5
*MdIAA32*	MD15G1169100	13036569 13038091	15	753	250	28.09	7.39	4
*MdIAA33*	MD15G1391700	48773621 48776944	15	1,092	363	39.35	6.92	6
*MdIAA34*	MD16G1227100	23027760 23030866	16	1,158	385	40.61	8.82	5

**Note:**

ORF, opening reading frame; MW, molecular weight; PI, isoelectric point.

### Phylogenetic relationships of Aux/IAA proteins

**Figure 1:**
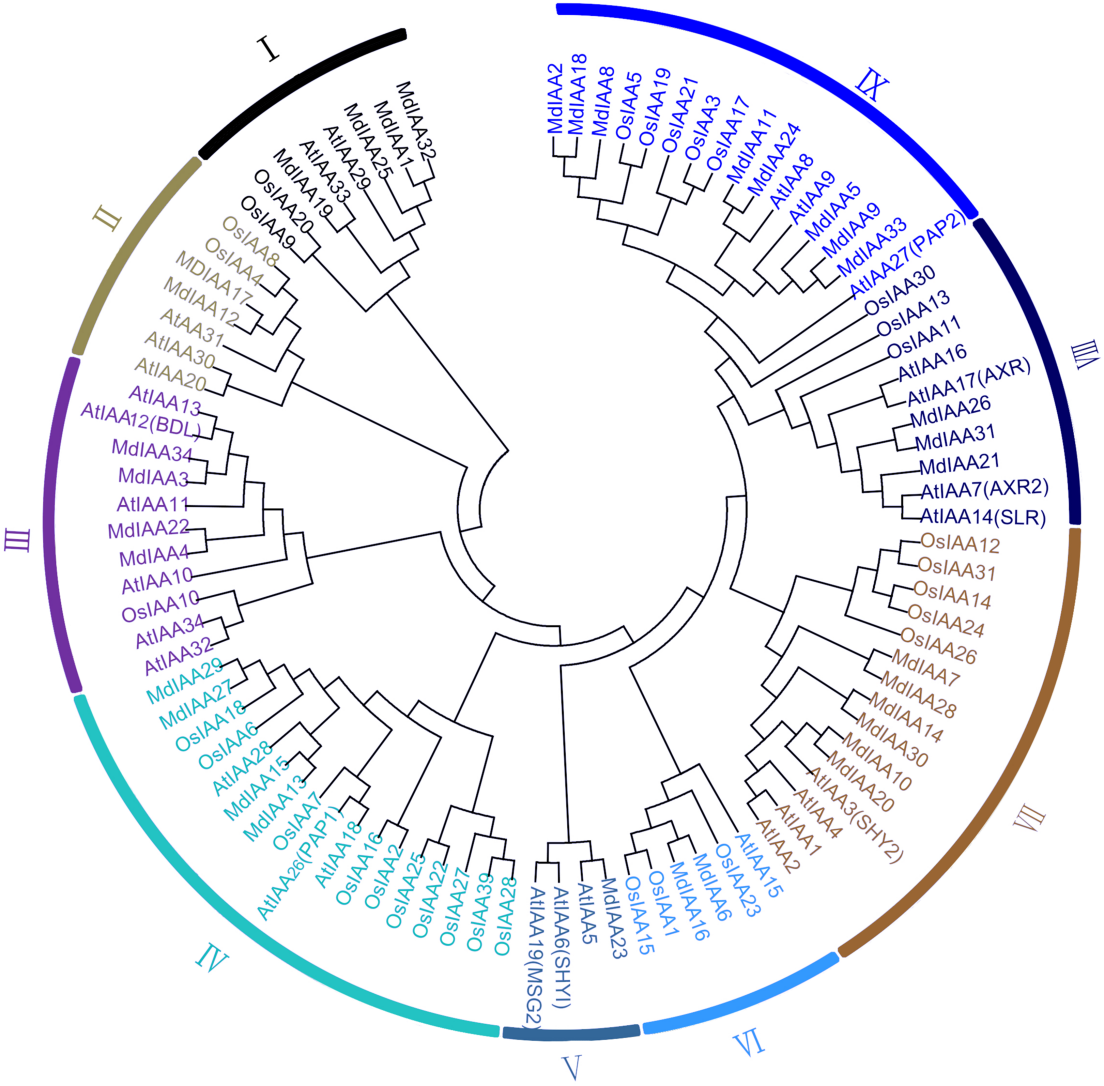
Phylogenetic analysis of 99 AUX/IAA proteins from apple, rice, *Arabidopsis*. The phylogenetic tree was generated using MEGA 6.0 with the Neighbor-Joining (NJ) method with 1,000 bootstrap replicates. Aux/IAA members of the three species could be split into nine subgroups, which were numbered I–IX.

A phylogenetic tree was constructed which included 34 members in apple, 34 members in *Arabidopsis*, and 31 members in rice. According to their phylogenetic relationships, Aux/IAA members of the three species could be split into nine subgroups, which were numbered I–IX (Fig. 1). The number of MdIAAs in the nine subgroups ranged from four to 17. The 34 MdIAAs were distributed unevenly among the subgroups. The numbers of MdIAAs in Subgroups I, II, III, IV, V, VI, VII, VIII, and IX were 4, 2, 4, 4, 1, 2, 6, 3, and 8, respectively.

### Sequence alignment, gene structure, and conserved motifs of Aux/IAA proteins in apple

**Figure 2:**
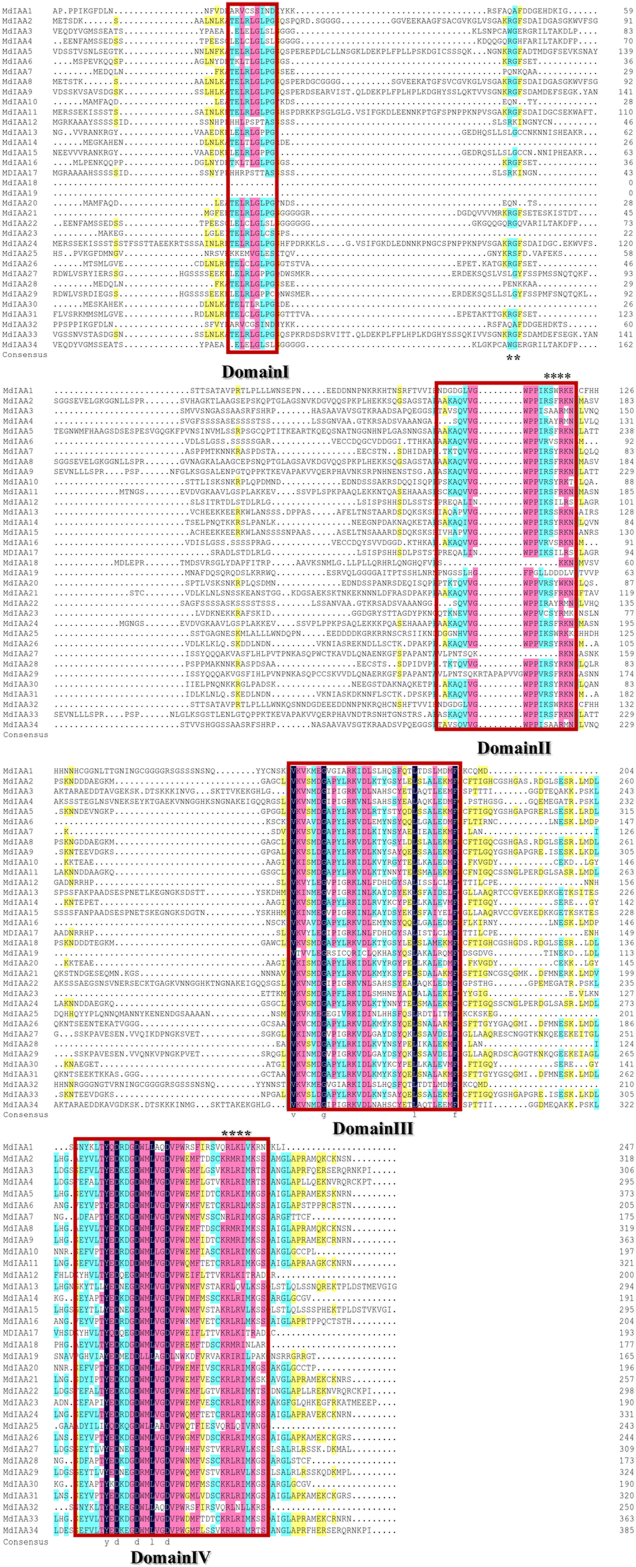
Amino acid sequence alignment and domain analysis of MdIAA family proteins. Multiple alignments were conducted for amino acid alignment and domain analysis among the MdIAA family proteins by using the DNAMAN program. The similar amino acids among the proteins were highlighted with different colors. Four domains of MdIAA proteins were marked with red frames. Nuclear localization signals (NLS) are indicated with black asterisks.

There were four conservative domains from a sequence alignment of the ORF translation of the Aux/IAA genes (Fig. 2). Nuclear localization signals were also found in most MdIAAs. Amino acids at five positions were absolutely conserved among all MdIAAs, but amino acids in 30 positions were strongly conserved (75–99%). The similarity between the other amino acids was <50%.

**Figure 3:**
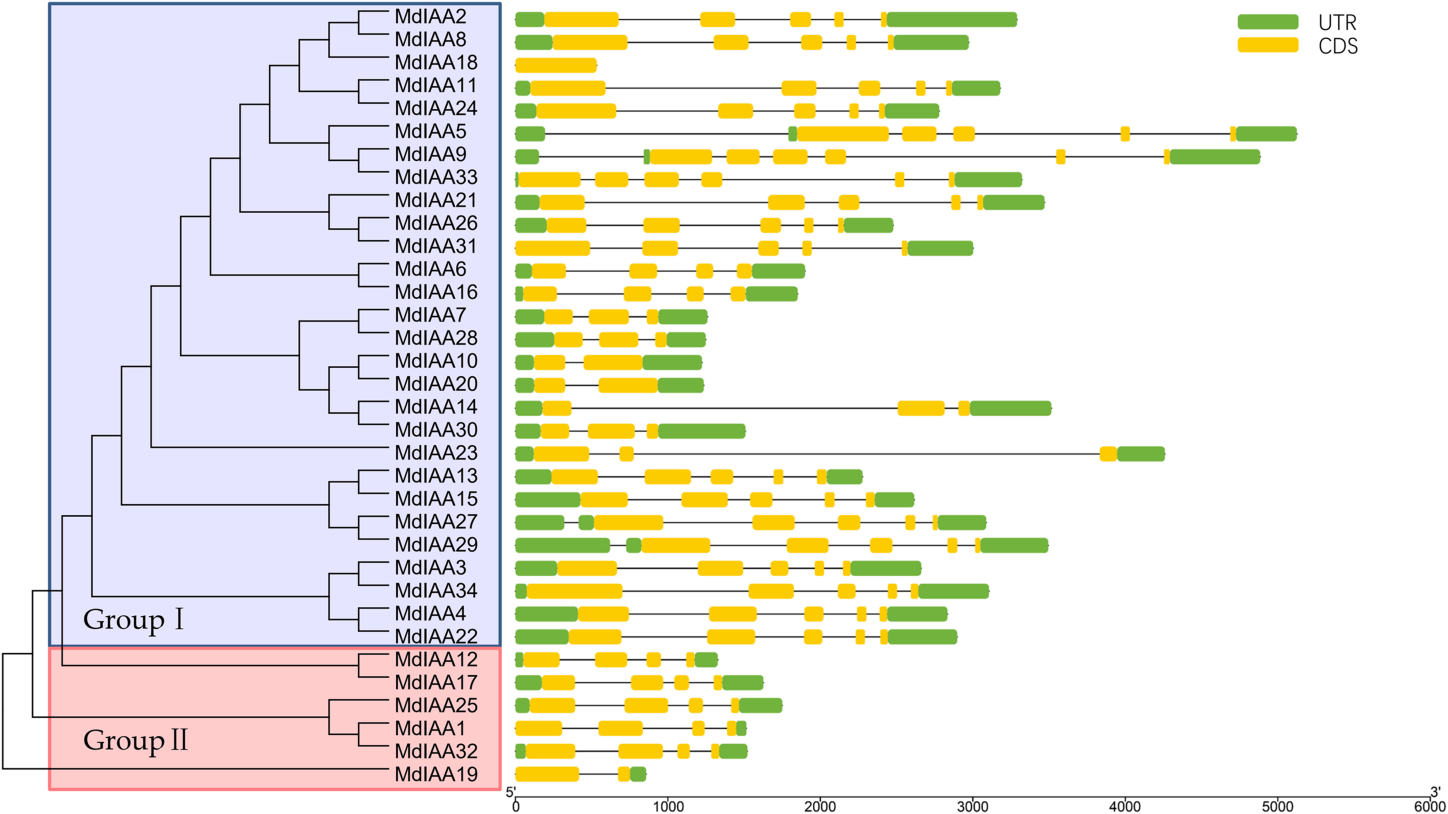
Phylogenetic relationships and structures for 34 MdIAAs. The phylogenetic tree for full-length amino acid sequences was constructed with MEGA software and the NJ method. Aux/IAA members of apple could be split into two subgroups, which were numbered I and II. The exons and introns are represented by green boxes and lines, respectively. The 5′- and 3′-untranslated regions (UTRs) are represented by yellow boxes.

To explore the *MdIAA* gene’s structural diversity in apples, we evaluated the conservation of the exon-intron organization. The MdIAA proteins were divided into groups I and II according to their phylogenetic relationships (Fig. 3). The gene structure analysis suggested that in group I, although *MdIAA18* had one exon and the other two genes (*MdIAA9* and *MdIAA33)* had six exons, most genes had four exons. In group II, only *MdIAA19* contained two exons and the remaining genes contained four exons.

**Figure 4:**
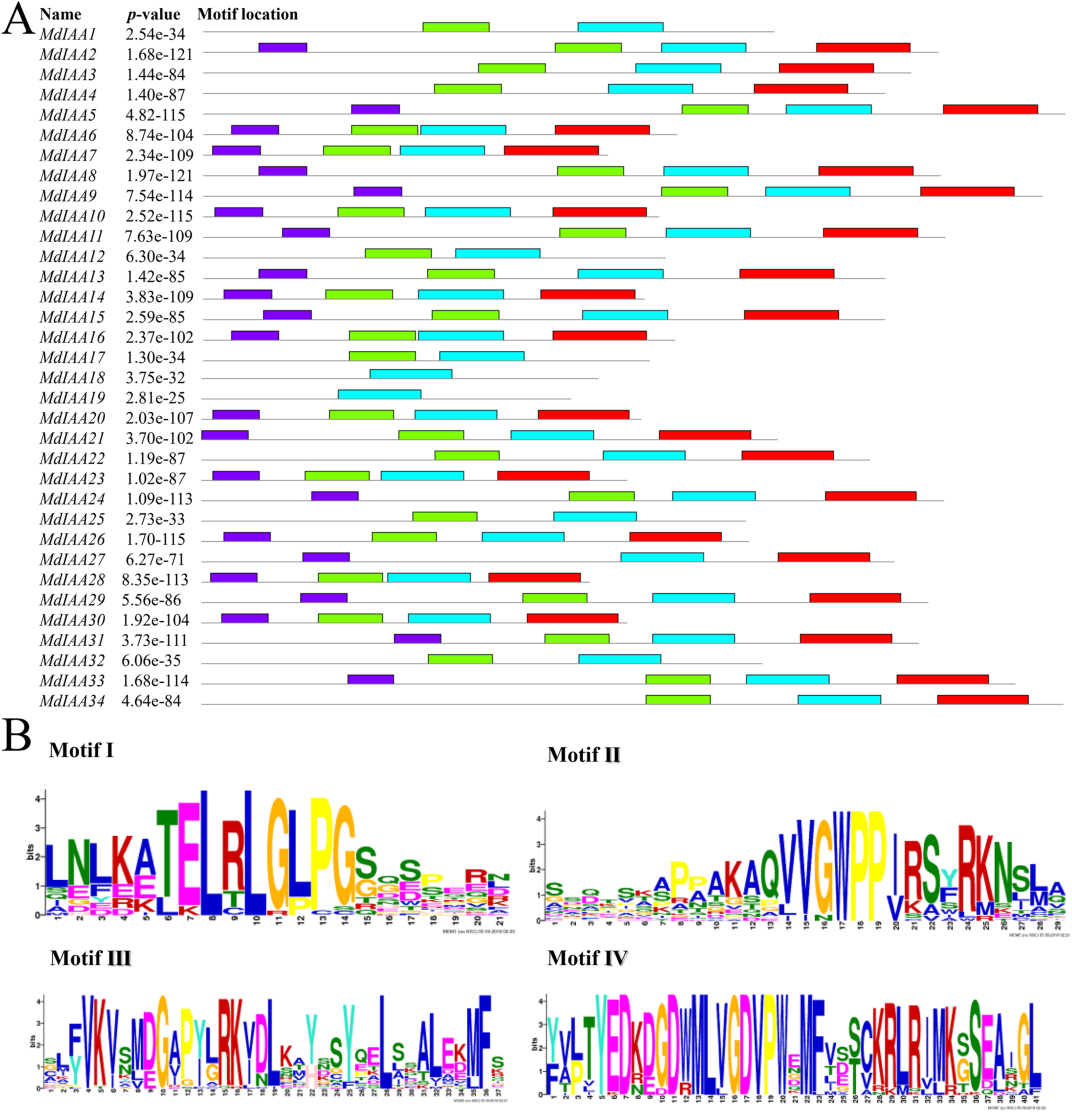
The motif distribution of the MdIAA proteins. (A) Shows 4 classical motifs I, II, III, and IV (labelled by different color blocks) of the Aux/IAA proteins, which were analyzed by the Multiple Expectation Maximization for Motif Elicitation (MEME) web server. (B) The degree of specific amino acids conservation in each motif of Aux/IAA varies with the height of each letter.

We identified common motifs using the MEME web server (http://meme-suite.org/tools/meme) (Fig. 4). The following parameter settings were used: size distribution, zero or one occurrence per sequence (zoops); motifs count, 4; and motif width, between six and 50 wide. In our research, the four motifs identified by MEME were consistent with the four classical domains. This identified four motifs, which corresponded to the four conserved domains found through sequence alignment above. All four motifs were apparent in 22 of the proteins, although only three motifs were found in seven additional proteins. Motif I was not contained in MdIAA3, MdIAA4, MdIAA22, and MdIAA34; Motif IV was not in MdIAA12 and MdIAA4; and Motif II was not in MdIAA27. Three MdIAAs, MdIAA1, MdIAA25, and MdIAA32, contained only Motif II and Motif III, and two other MdIAAs, MdIAA18 and MdIAA19, contained only Motif II. It is obvious that most of the domains and motif regions overlapped with each other. Additionally, several highly conserved key sequences, such as LxLxLx in Domain I and Motif I, and VGWPP in Domain II and Motif II, strongly suggests that these regions may be the key sites conferring the functions of MdIAAs.

### Cloning of apple *IAA* genes

To confirm the coding sequence of MdIAAs as annotated in the reference genome and to further uncover the biological function of these MdIAAs, 17 *MdIAA* genes were cloned: *MdIAA4*, *MdIAA5*, *MdIAA8*, *MdIAA9*, *MdIAA10*, *MdIAA14*, *MdIAA15*, *MdIAA1*6, *MdIAA20*, *MdIAA21*, *MdIAA24*, *MdIAA26*, *MdIAA27*, *MdIAA28*, *MdIAA30*, *MdIAA31*, and *MdIAA33*, respectively. As a result, all cloned sequences showed a high similarity to the predicted apple genome (Data S1). The sequences were submitted to NCBI GenBank.

### Analysis of promoter cis-elements of apple *IAA* genes

**Figure 5:**
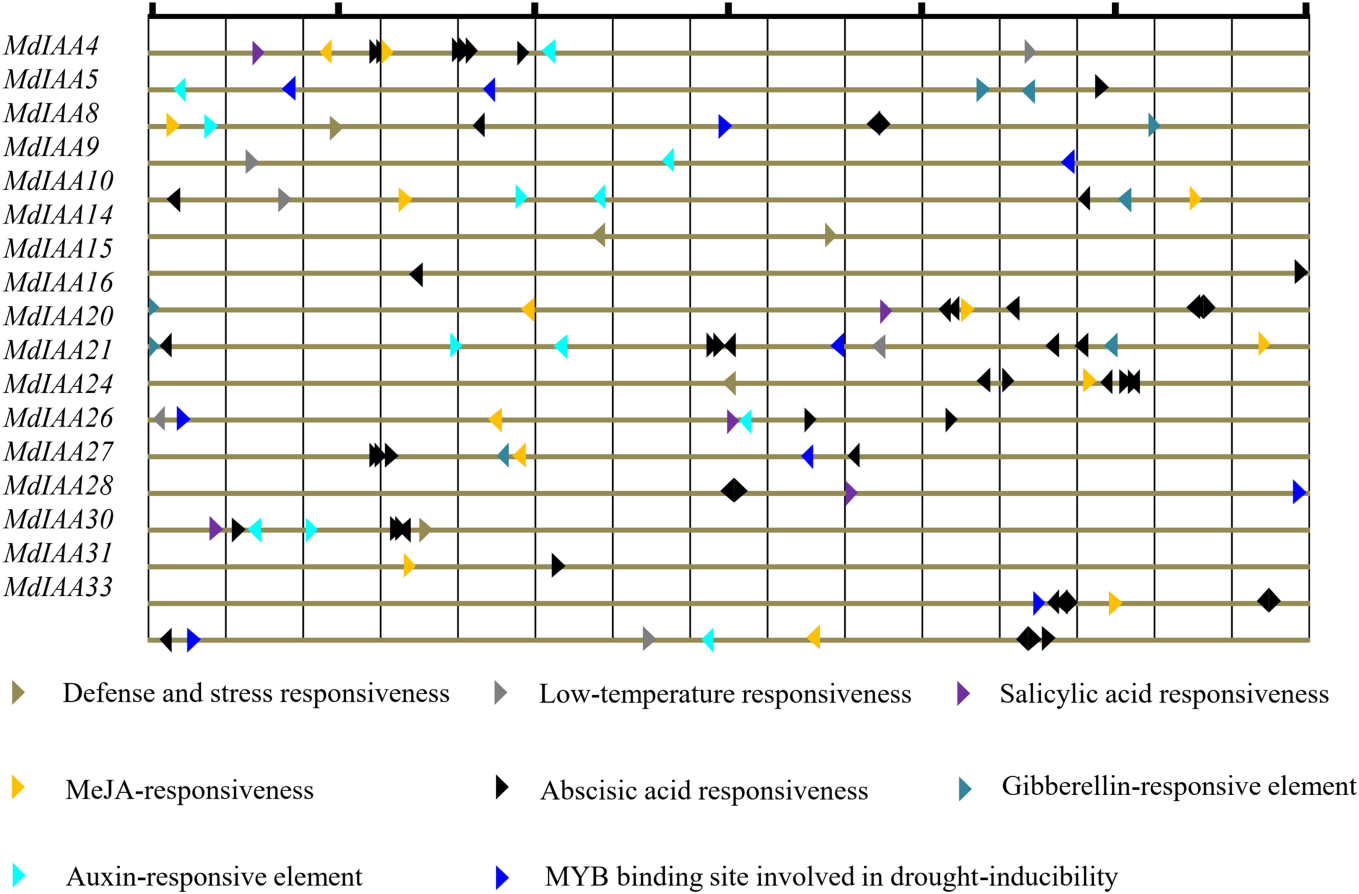
The main putative cis-elements within 1.5 kb upstream promoter regions of *MdIAAs*. The different cis-elements are shown by using different icons and colors. The direction of the apex angle to the right indicates the cis-acting element in the (+) strand, and the direction of the apex angle indicates to the left the cis-acting element in the (−) strand.

Promoter cis-regulatory elements are of great significance in regulating gene expression. In this study, the putative cis-elements within the *MdIAA* promoter (<1.5 kb upstream from the start site of putative translation) were identified through the PlantCARE database (Fig. 5) and different kinds of cis-elements related to stress and hormone responses were identified. Most *MdIAA* promoters contained ABRE elements, which usually participate in ABA related responses. Nine of the *MdIAA* genes contained a TGA-box, which is an auxin-responsive element. Six of the *MdIAA* genes contained gibberellins-responsive elements such as P-box, GARE-motif or TATC-box. A total of 11 of the *MdIAA* genes contained a G-box, CGTCA-motif, and/or TGACG-motif, which participate in JA/MeJA-responses. Five of the *MdIAA* genes contained a TCA-element, which is related to salicylic acid responsiveness. A large number of putative hormone-responsive elements suggested that these genes were involved in hormone signal transduction. Moreover, the promotors contained drought-inducibility elements (MBS), low-temperature response motifs (LTR), and defense and stress responsiveness elements (TC-rich repeats). The results indicated that *MdIAA* genes may be involved in defense signaling and biotic and abiotic stress in apple plants.

### The spatio-specific profiles of MdIAA genes expression in various tissues

**Figure 6:**
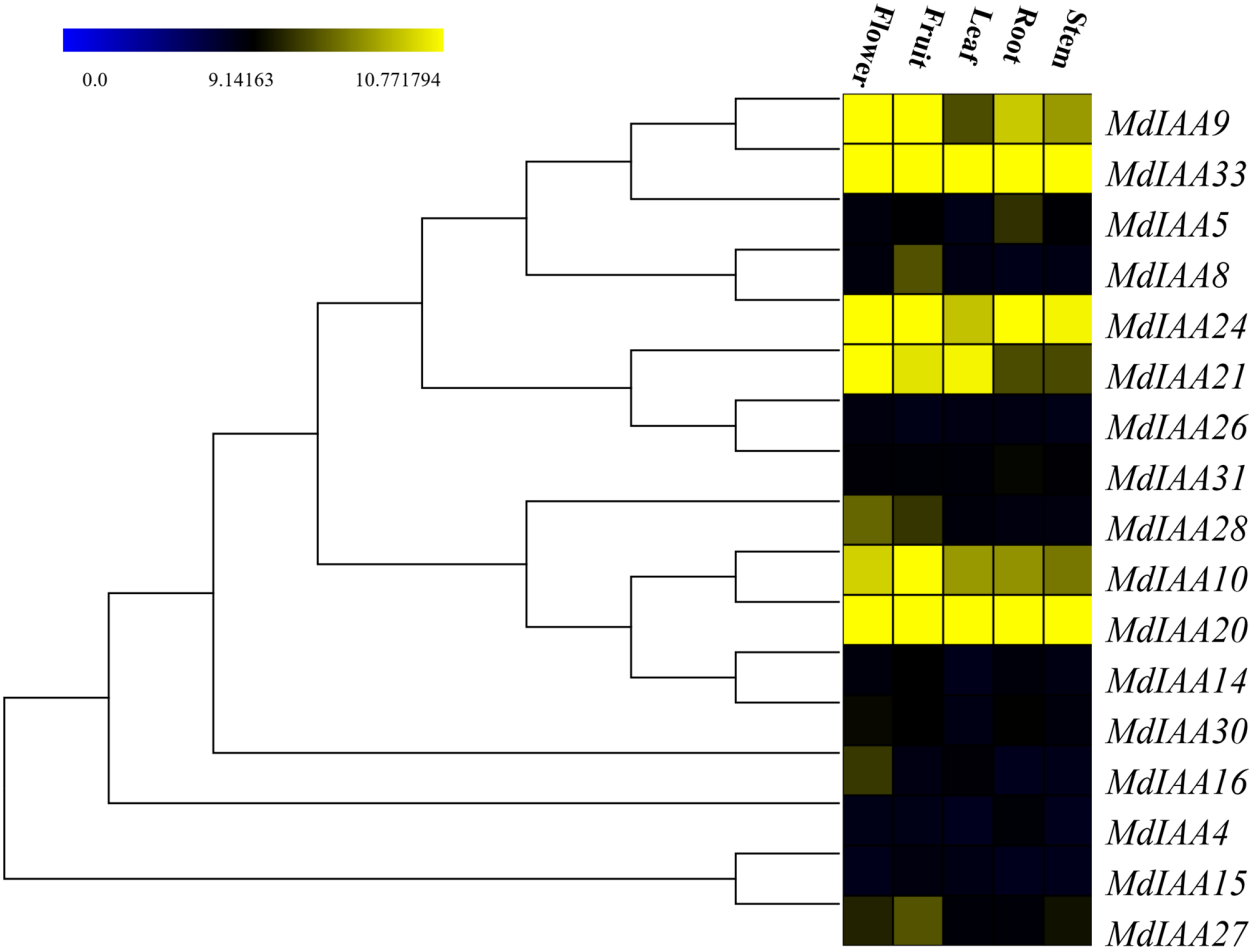
Expression profiles of *MdIAA* genes in various tissues. Heat map of *MdIAA* genes expression in flowers, fruit, leaves, roots, and stems of apple plants generated by using TIGR MeV v4.8.1 software. Levels of down-regulated expression (green) or up-regulated expression (red) are shown on a log^2^ scale from the highest to the lowest expression for each *MdIAA* gene.

In order to determine the additional roles that *MdIAAs* play in growth and development, 17 selected *MdIAA* genes were examined in the roots, stems, leaves, flowers, and fruits using qRT-PCR. Some *MdIAAs* showed higher expressions in multiple tissues (Fig. 6). For instance, there were high levels of expression of *MdIAA20* and *MdIAA33* in all of the examined tissues. The expression of *MdIAA20* was the highest in flowers and fruit while the expression of *MdIAA33* was the highest in the roots, stems, and leaves. In addition, a higher transcriptional accumulation in the flowering and fruiting stages of *MdIAA4*, *MdIAA9*, and *MdIAA24* were found, suggesting that they may play specific roles in the development of flowers and fruit. The mRNA abundances of the MdIAA family genes were higher in fruit than in other tissues. Furthermore, the expression of *MdIAA* genes in the stems was the lowest among all the tissues. These results indicated that *MdIAA* genes might play roles in fruit. Overall, the tissue-specific spatial differential expression of *MdIAA* suggests that these genes were specifically related to apple growth and development.

### Expression profiles of MdIAA genes following IAA treatment

**Figure 7:**
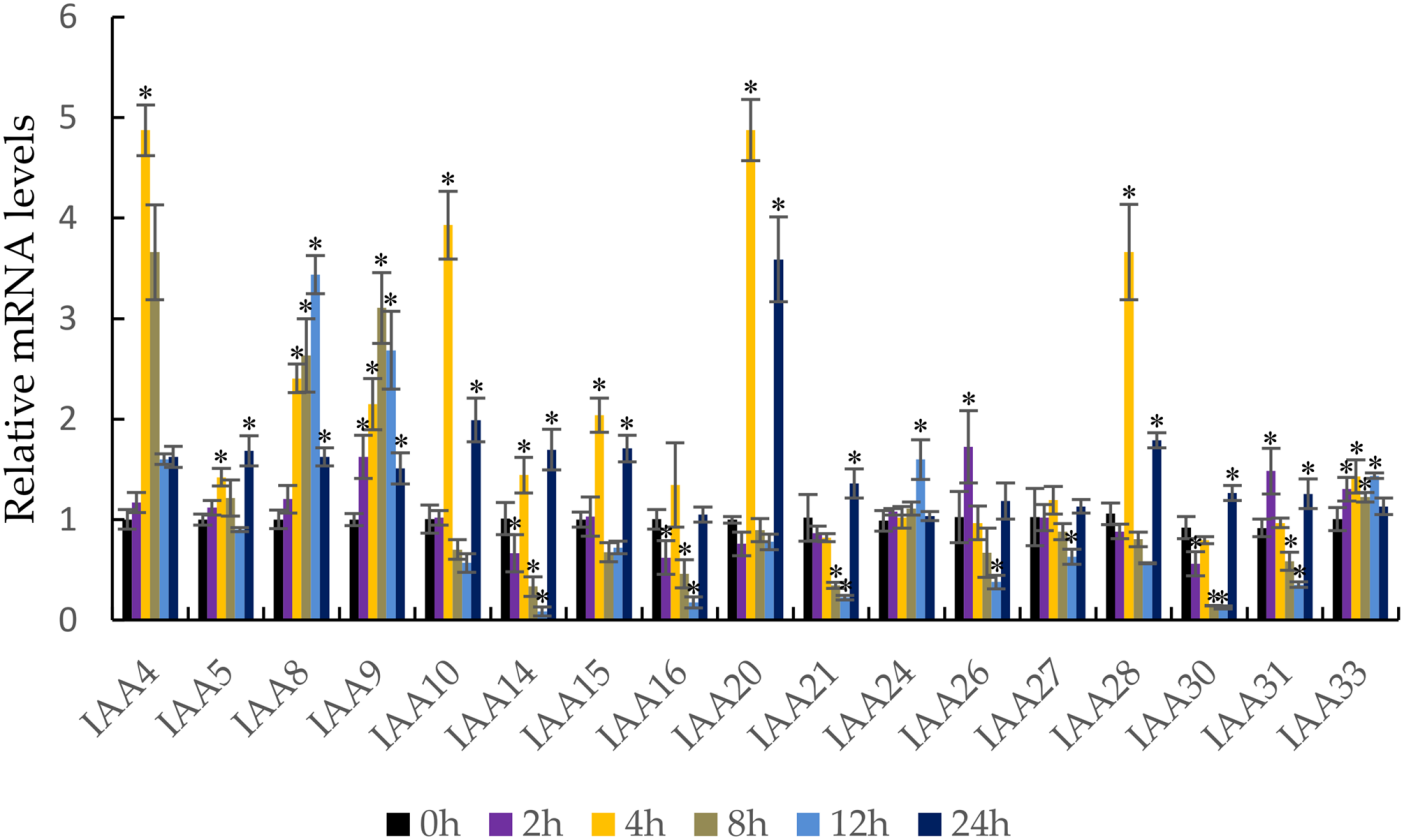
The expression patterns of *MdIAAs* in apple under auxin treatments. Samples were harvested at 0, 2, 4, 8, 12, and 24 h after foliar application. Error bars show standard deviations of three biological replicates. Asterisks indicate a significant difference (*P < 0.05) compared with the corresponding controls, based on one-way ANOVA and Tukey’s tests.

It is well known that auxin induces the strong expression of the *Aux/IAA* genes (Liu et al., 2017). qRT-PCR was performed to confirm the response of the *MdIAA* genes following IAA treatment. In this work, five testing time points (0, 2, 4, 8, 12, and 24 h) were selected. Under IAA treatment, the *MdIAA* family genes displayed various expression styles. The expressions of the majority of the *MdIAA* genes were up-regulated at 4 h as compared with controls (Fig. 7), but the expressions of most *MdIAA* genes were down-regulated at 8 and 12 h as compared with controls. No significant changes in gene expression were observed in other genes, such as *MdIAA27*, after IAA treatment. Taken together, this data indicated that most genes from the *MdIAA* family were auxin early-response genes.

### The profiles of MdIAA gene expression following abiotic stresses and ABA spray

**Figure 8:**
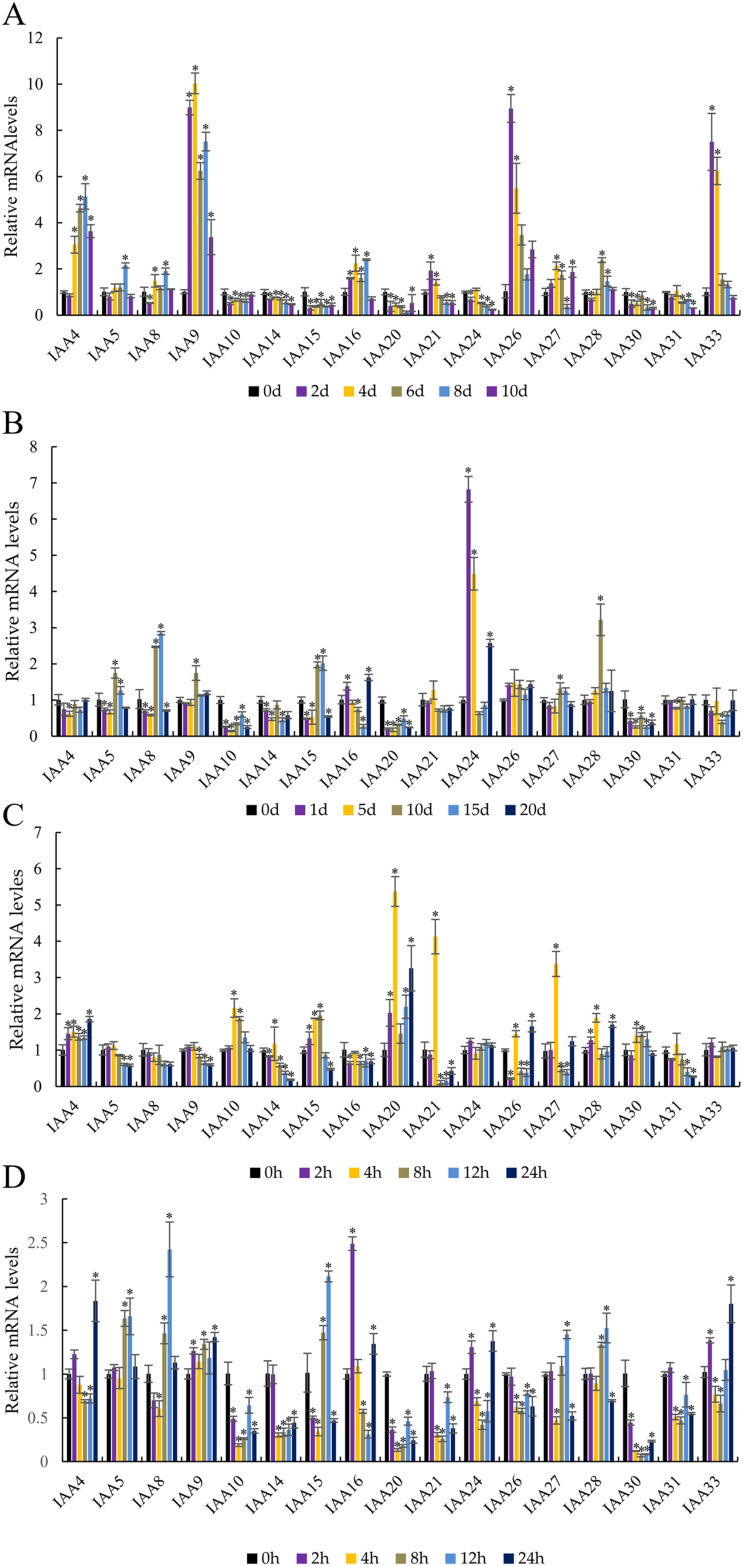
Abiotic stress altered expression of selected IAA genes in apple leaves. (A) Drought: samples were collected at 0, 2, 4, 6, 8, and 10 days after withdrawal irrigation. (B) NaCl treatment: samples were harvested at 0, 1, 5, 10, 15, and 20 days after irrigation with 200 mM NaCl solution. (C) Chilling challenge: samples were harvested at 0, 2, 4, 8, 12, and 24 h after exposure to 4 °C. (D) ABA (100 µM): samples were harvested at 0, 2, 4, 8, 12, and 24 h after foliar application. Error bars show standard deviations of three biological replicates. Asterisks indicate a significant difference (*P < 0.05) compared with the corresponding controls, based on one-way ANOVA and Tukey’s tests.

To investigate the effects of abiotic stresses and ABA treatment on MdIAA gene expression, the patterns of transcription were observed after drought, cold, NaCl, and ABA treatment (Fig. 8). Under drought stress, the expressions of *MdIAA* genes showed diverse responses at different time points (Fig. 8A). Among the 17 *MdI*AA selected genes, *MdIAA4*, *MdIAA26*, and *MdIAA33* increased significantly. The expression of *MdIAA4* was 5.15 times that of the control group at 8 days of drought treatment and the expression of *MdIAA26* was 8.95 times that of the control group at 2 days and 5.50 times that at 4 days of drought treatment, respectively. The expression levels of *MdIAA33* showed a trend similar to *MdIAA26*, being 7.50 times and 6.24 times that of the control group at 2 and 4 days, respectively. However, in this study, the expression levels of several *MdIAA* genes were significantly lower than those of the control group after drought treatment. For instance, the expression of *MdIAA20* was only 12.7% of the control group at 8 d under drought treatment, while the expression of *MdIAA24* was only 23.8% of the control group at 10 days under drought treatment.

Two genes showed an obvious response to the NaCl treatment (Fig. 8B). The expression of *MdIAA24* was 6.82 and 4.49 times that of the control group at 1 and 5 days, respectively, but the expression of *MdIAA28* was 3.22 times that of the control group at 15 days. However, some *MdIAA* gene expression was dramatically reduced after NaCl treatment. For example, the expression of *MdIAA10* and *MdIAA20* was only 14.1% and 17.7% of the control group, respectively.

In response to chilling at 4 °C, three *MdIAA* genes, *MdIAA20*, *MdIAA21*, and *MdIAA27*, increased significantly at 4 h of treatment (Fig. 8C). However, their expressions were then down-regulated over time. For example, at 8 h, *MdIAA21* was only 10.82% of the control group. The other genes, such as *MdIAA24* and *MdIAA33*, showed no alterations in expression under most conditions.

After ABA application (Fig. 8D), the expressions of *MdIAA10*, *MdIAA14*, *MdIAA20*, *MdIAA21*, and *MdIAA30* decreased significantly. Among these genes, *MdIAA30* changed most dramatically, and was only 7.05% of the control group at 8 h although some genes increased significantly during this time. For instance, the expression of *MdIAA25* was 2.50 times that of the control group at 2 h whereas *MdIAA8* was 2.42 times that of the control group at 12 h. Hence, these genes were designated as being ABA-responsive.

### Overexpression of MdIAA9 enhanced plant growth of tobacco seedlings under osmotic stress

**Figure 9:**
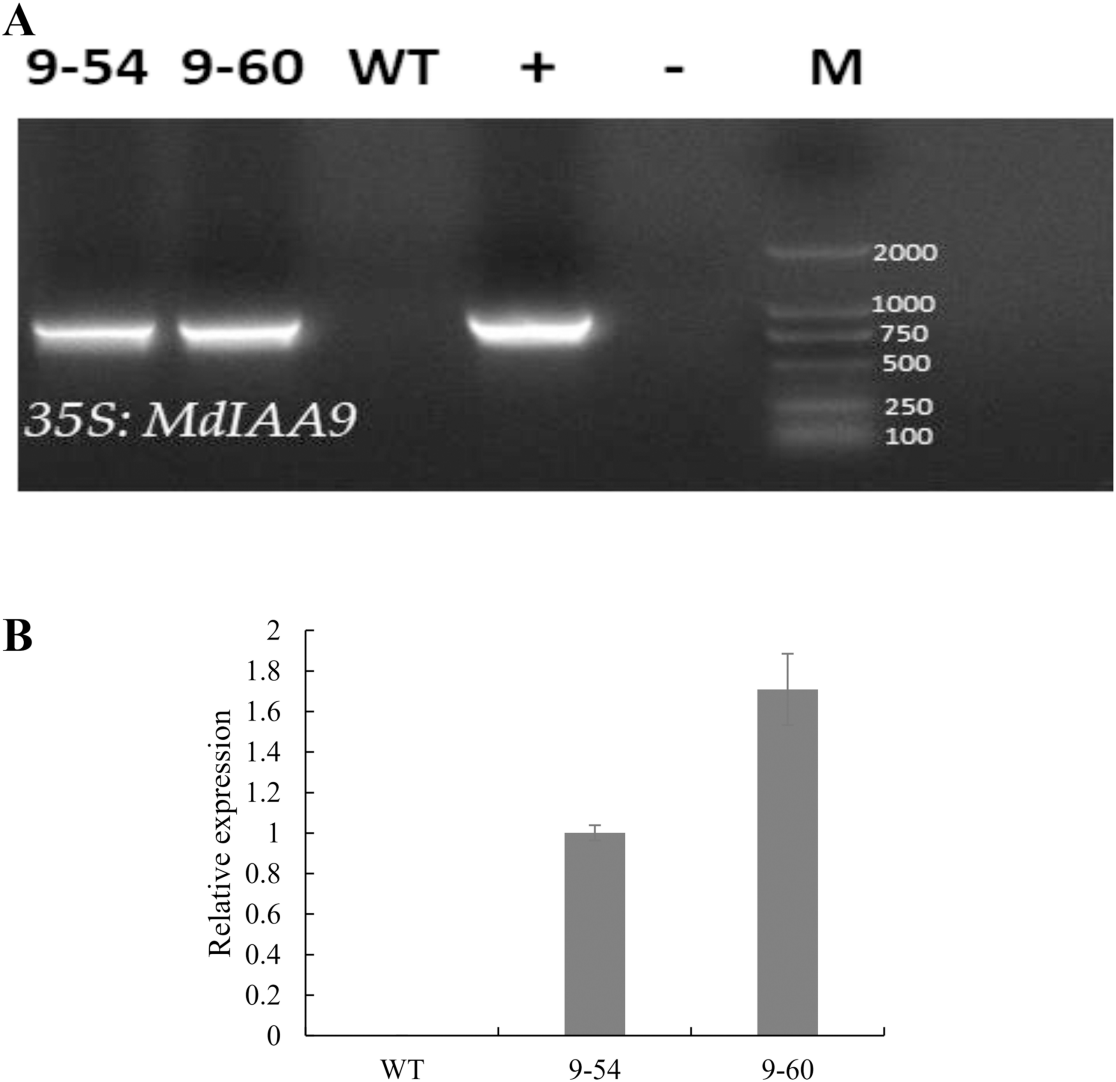
PCR identification and relative expression analysis of *MdIAA9* in wild-type and transgenic *tobacco* lines. (A) *35S* and *MdIAA9-R* indicate that the primers used to identify the transgenic lines were designed according to the *MdIAA9*. M, DNA marker; −, negative control (H_2_O); +, positive control (plasmid DNA of *35S::MdIAA9* pBI121 vector). (B) Specific primers for *MdIAA9* were used to detect relative expression levels of WT and transgenic *tobacco* lines.

**Figure 10:**
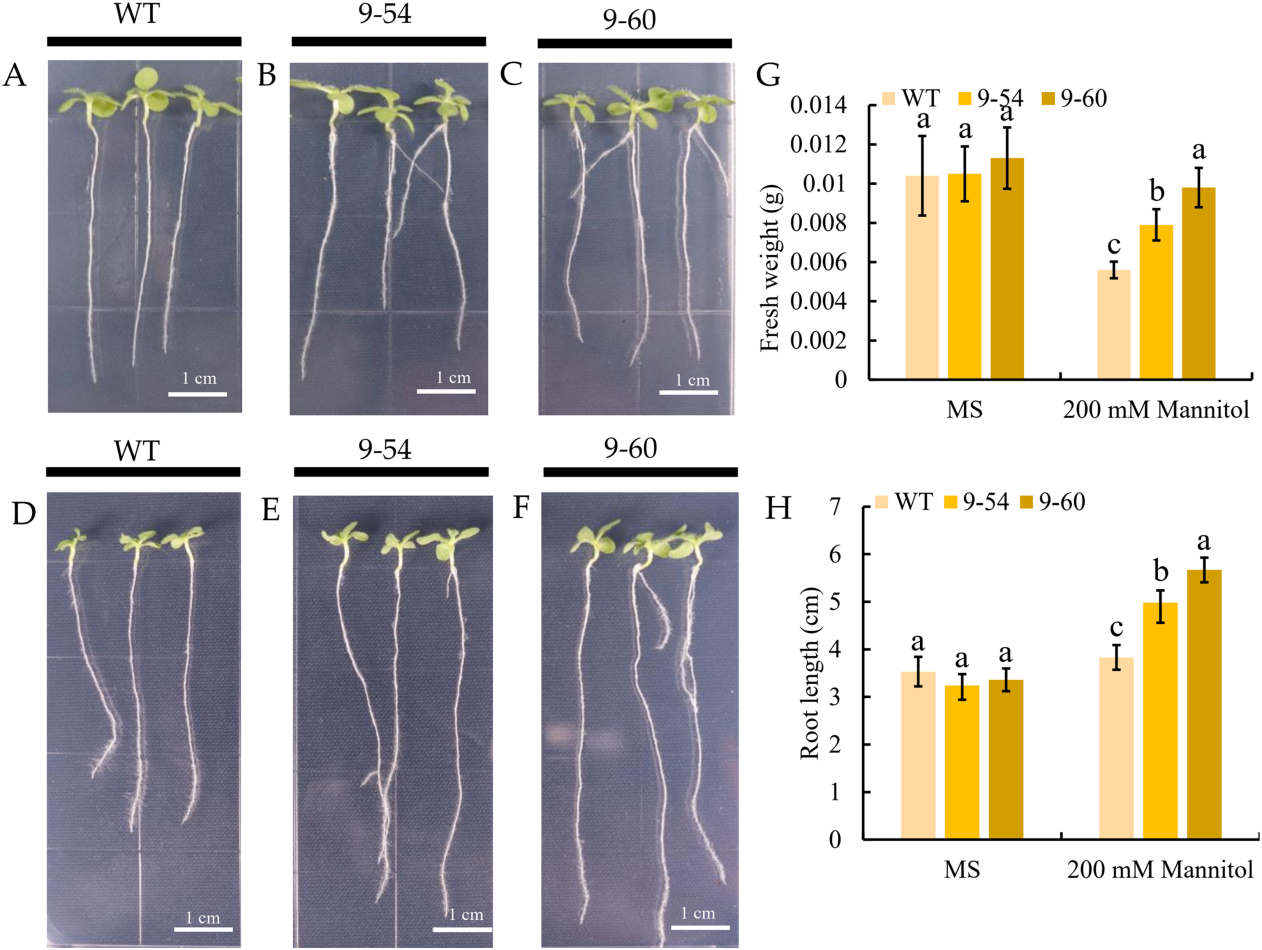
Comparative analysis of *MdIAA9*-overexpressing tobacco seedlings and wild type in response to mannitol treatment. (A–C) Representative images of wild type and *MdIAA9*-overexpressing seedlings grown vertically in MS medium contained zero mM mannitol for 13 days. (D–F) Representative images of wild type and *MdIAA9*-overexpressing seedlings grown vertically in MS medium contained 200 mM mannitol for 13 days. Bars = 1 cm. (G) Fresh weights between WT and transgenic lines after exposure to osmotic stress for 13 days. (H) Comparison of primary root lengths between WT and transgenic lines after exposure to osmotic stress for 13 days. Error bars represent standard deviations from three biological replicates. For (G) and (H) different letters that follow the values indicate significant differences between treatments at *P* < 0.05, based on one-way ANOVA and Tukey’s tests.

Under drought stress, *MdIAA9* was significantly up-regulated, so we chose this gene to study its biological function in tobacco. Two transgenic lines were identified at the DNA level, using 35S promoter positive primers and the reverse primers of *MdIAA9* (Table 2; Fig. 9). We examined the root length and fresh weights of transgenic and wild plants under osmotic stress. Our results showed that the root lengths and fresh weights of the transgenic plants were significantly higher than those of the wild types under osmotic stress. However, no significant difference was observed between the transgenic and wild plants under the control conditions (Fig. 10).

### Analysis of physiological characteristics of *MdIAA9*-overexpressing tobacco seedlings and wild type to osmotic stress

**Figure 11:**
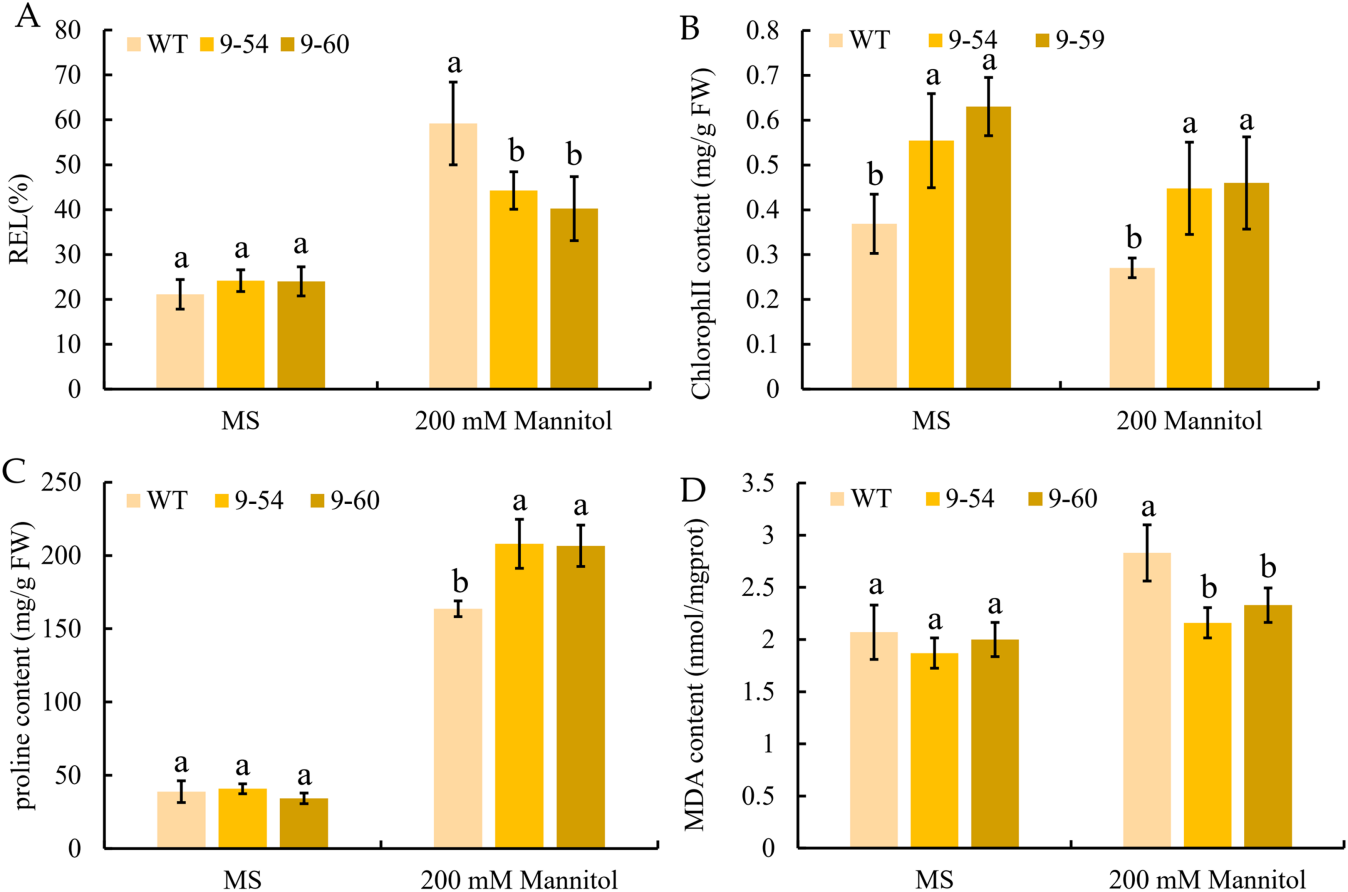
Response to osmotic stress in transgenic and wild tobacco seedlings. (A) relative electrolyte leakage (REL), (B) chlorophyll content, (C) proline content, and (D) MDA content in transgenic and WT seedlings after exposure to MS medium contained 0 or 200 mM mannitol for 13 days. Error bars represent standard deviations from three biological replicates. For (A–D), different letters following the values indicate significant differences between treatments at *P* < 0.05, based on one-way ANOVA and Tukey’s tests.

To further study the enhancement of osmotic stress tolerance mediated by *MdIAA9*, the REL, chlorophyll concentration, proline, and MDA concentrations were determined. These are important markers to measure the degree of osmotic stress injury. Under normal conditions, no significant difference was observed between the transgenic and wild plants. However, the REL values of the wild lines were significantly higher than the overexpressing tobacco seedlings under 200 mM mannitol conditions (Fig. 11A). The chlorophyll levels of wild and overexpressed plants were similar under both the osmotic stress and normal conditions. The quantities of proline in transgenic plants were significantly higher than the wild line under 200 mM mannitol, but no significant differences were revealed under normal conditions between the transgenic and wild plants (Figs. 11B and 11C). Furthermore, the MDA content of the transgenic plants was significantly lower than the wild plants under the 200 mM mannitol conditions, while no significant difference was observed in the transgenic lines and wild types under normal conditions (Fig. 8D). The results suggested that the OE of *MdIAA9* in tobacco seedlings leads to a higher tolerance of osmotic stress.

## Discussion

Indole-3-acetic acid, a key signaling molecule, plays a vital role in a series of processes during plant growth and development (Liu et al., 2015). Aux/IAA inhibits downstream gene expression through its interaction with ARFs (Liu et al., 2015; Yu et al., 2017; Kalluri et al., 2007). Plants are subjected to environmental stresses such as drought, salinity, and low temperature, adversely affecting their growth and development (Tan et al., 2014). Auxin-responsive genes are thought to participate in various responses related to environmental stress (Guo et al., 2018; Shani et al., 2017).

Many family members of Aux/IAA genes have been identified to date from different plant species such as *Arabidopsis*, tomato, rice, *Medicago*, and papaya (Yuan et al., 2018; Wang et al., 2010b; Shen et al., 2014; Wu et al., 2014; Jain et al., 2006). The characterization and expression analysis of the *MdIAA* genes help investigators to further understand the involvement of auxin in various types of stress. However, in the apple, there is little known about *IAA* genes or their expression. A total of 34 *MdIAA* genes were identified in this study, which are as many as have been identified in *Arabidopsis*.

We found that most of the MdIAA proteins possessed four classically conserved domains. The comparison of those conserved domains showed that 10 MdIAA genes (MdIAA1, MdIAA3, MdIAA4, MdIAA12, MdIAA17, MdIAA18, MdIAA19, MdIAA22, MdIAA25, and MdIAA34) lacked Domain I, which acts as a repressor in the signaling pathway of auxin. The MdIAA proteins contained Domain II (except for MdIAA18, MdIAA19, and MdIAA27), which was necessary for the protein degradation process mediated by auxin (Dharmasiri, Dharmasiri & Estelle, 2005). It is noteworthy that all MdIAA proteins contained Domain III and most of MdIAA proteins contained Domain IV. Our results suggested that most MdIAAs might interact with ARFs by forming stable homo- and hetero-dimers.

The *Arabidopsis* and rice *Aux/IAA* gene families have been well characterized. Useful information will be provided about the biological functions of the apple through comparative studies on phylogenetic relationships. We concomitantly monitored the expression patterns of 17 *MdIAA* genes from different apple tissues. Plants floral initiation is an important stage in their life cycle. In *Arabidopsis*, the gain-of-function mutant *IAA7/AXR2* confers delayed flowering under short-day light (Mai, Wang & Yang, 2011). Interestingly, *MdIAA21*, a homologous gene of *AtIAA7*, was highly expressed in flowers (Fig. 4), which suggested that *MdIAA7* might play an important role in the apple flowering process. However, *AtIAA14* proved to have an important role in the development of both lateral and adventitious root development (Lopez-Bucio et al., 2015). The orthologous gene of *AtIAA14* in apple, *MdIAA21*, was preferentially expressed in the flowers, which indicated that the apple IAA family genes may vary in the evolution for their own growth and development. Furthermore, a phylogenetic tree was constructed to reveal the evolutionary relationships of IAA genes between apple and rice. Interestingly, in rice, the *OsIAA11* gene was reported to play an essential role in the development of the root system (Zhu et al., 2012; Ni et al., 2014). According to the phylogenetic tree analysis, the homologous gene of *OsIAA11* in the apple was *MdIAA21*, which was preferentially expressed in flowers. This suggested that the *MdIAA* genes might not perform the same biological functions as the IAA genes in other already reported species.

As an important primary auxin-responsive gene family, auxin treatment rapidly induces the expression of the IAA family. According to the results of the IAA treatment, 17 apple IAA genes were selected and their expression patterns were studied. The expression of *MdIAA* genes was either higher or lower than the control after different amounts of exposure to IAA treatment. We concluded that the treatment time of IAA played a crucial role in the up-regulation or down-regulation of auxin responsive genes.

Extensive studies have shown that the changes in auxin concentration, redistribution, and signal transmission are affected by various integrated environmental factors (Shibasaki et al., 2009). Mounting evidence shows that the auxin-response genes, including *Aux/IAA*, *GH3*, and *SAUR* are related to stress and defense responses in *Arabidopsis*, maize, rice, and *Sorghum bicolor* (Ghanashyam & Jain, 2009; Jain & Khurana, 2009; Zhang et al., 2009; Wang et al., 2010a). In our research, *MdIAAs* showed various expression patterns under different stress conditions. The expression levels of some *MdIAAs* increased after drought, cold, salt, and ABA exposure. However, the expression levels of some *MdIAAs* decreased after treatments. Similar results have been shown in other species. In the tomato, the up-regulated expression of most *SlIAAs* under drought and salt stress treatments was observed, while the expression levels of *SlIAA3*, *SlIAA11*, and *SlIAA14* decreased after stress treatments (Wu et al., 2012). Here, drought-inducibility elements (MBS), LTR motifs, and defense and stress responsiveness elements (TC-rich repeats) were shown to be upstream of the promoter regions in some *MdIAAs*. These cis-elements related to abiotic stress and auxin signaling regulating pathways might result in different expression patterns of *MdIAA* genes. However, the results from qRT-PCR analysis were inconsistent with the analysis of the promoter region on *MdIAA* genes. For instance, although some LTR motifs were found in *MdIAA24* and *MdIAA33*, no significant alterations in their expression levels were observed under the cold conditions and conversely, in the promoter regions of *MdIAA10* and *MdIAA20*, no cis-elements related to salt stress were found. However, qRT-PCR analysis showed that the two genes in question were significantly responsive to stress. This indicated that the expression of those MdIAAs in stress response might be regulated by some unidentified cis-regulated elements in the apple.

In the present study, as compared with the wild type, the OE of *MdIAA9* in tobacco seedlings increased their root lengths and fresh weights. Furthermore, this OE also induced a series of changes in tobacco seedlings related to abiotic stress responses, including REL, total chlorophyll concentration, free proline, and MDA content. The lower levels of REL and MDA and increased levels of proline and total chlorophyll in the overexpressed lines indicated that *MdIAA9* has a significant role in enhancing resistance to osmotic stress. The present study indicated that OE of *MdIAA9* in tobacco seedlings led to an enhancement of drought resistance.

## Conclusions

A total of 34 *MdIAA* genes were identified and characterized in this study. A comprehensive *MdIAA* gene family genome-wide analysis was presented, which included the phylogeny, chromosome locations, gene structures, and conserved motifs. Our research shows that most members of the *MdIAA* gene family have different patterns of spatio-temporal transcript accumulation. Exogenous auxin can induce *MdIAAs* at the transcriptional level, most of which may be induced by exposure to drought stress, salt, cold, and ABA in the apple. Our research also shows that the OE of *MdIAA9* in tobacco seedlings leads to an enhanced drought resistance. The comprehensive information from the results enhances our recognition of how *MdIAA* genes function at different points in the life cycle and under various abiotic stresses. Notably, the present study implies that the stress-responsive genes may be appropriate candidates to create stress-tolerant apple rootstocks and cultivars using transgenic technology.

## Supplemental Information

10.7717/peerj.7935/supp-1 Supplemental Information 1 The 17 IAA gene sequences of apple.

10.7717/peerj.7935/supp-2 Supplemental Information 2 Raw Data.

## Additional Information and Declarations

### Data Availability

The following information was supplied regarding data availability: The raw data for all the figures is available in a Supplemental File.
