# Supplementary material for: Overexpression of MdIAA9 confers high tolerance to osmotic stress in transgenic tobacco
Source: PeerJ. 2019 Oct 31;7:e7935. doi: 10.7717/peerj.7935 (PMC6825743; doi:10.7717/peerj.7935)
Supplement: Supplemental Information 1 [file peerj-07-7935-s001.docx]

**Supplementary Data S1: The 17 IAA gene sequences of apple.**

>MdIAA4

ATGGAGAGTGGAGGTTCTGCAAGTGGGTCGTTGACTAAGTCAACGCTGTCCAGGGAGGAGAATTTTGCCATGTCTTCTGAGGACTCTTCTACCCCTGAGGAGTCTGGCCTCGAGCTCTGTCTTGGGCTGAGCCTTGGTGGTGGTGGAGGGAAGGATCAGCAGGGGCAAAGGGGTCACTTTGCGAGGATCTTGACTGCTAAGGATTTTCCTTCTGTGGGGTTTTCTTCTTCGTCGGCTTCCGAGTCGTCGTCTTCTACTTCTTCGTTGAGCAGCGGTAATGTTGCTGCTGGAACCAAGAGAAGTGCTGATTCTGTGGCTGCTGCTAATGGTGCCAGTCAGGTTGTGGGATGGCCTCCTATCAGAGCTTATAGAATGAATAGTTTGGTTCTCCAAGCGAAATCTTCATCCACTGAAGGACTTAACTCAGTAAATGAGAAAAGTGAATACAAGACCGGTGCAGAGAAGGTTAACAATGGAGGTCACAAGTCCAATGGAAATGCTAAGGAGATAGGGCAGCAGAGGGGTTCACTGTTTGTGAAGGTGAATATGGACGGGATTCCTATTGGGCGAAAGGTTAATCTAAGTGCGCATAGTTCCTATGAAGCGCTAGCACAAAAATTGGAGGATATGTTTGGGCCCT

CGACACATGGTTCAGGTGGTCAGGAGATGGAAGGAGCAACAAGACCCTCAAAGTTGCTGGATGGATCATTTGAGTTTGCGCTCACTTATGAAGATAAAGATGGAGACTGGATGCTTGTGGGAGATGTTCCTTGGGAGATGTTTCTTGGTACTGTCAAGAGGTTAAGGATTATGAGGACATCTGAGGCTAACGGACTTGCTCCCCTGTTACAGGAAAAGAATGTGAGGCAAAGATGTAAGCCAACCTAG

>MdIAA5

ATGTCTCCACCACTATTGGGTGGTGAGGAGGAAGGGCAGAGCAATGGGTCTATGGTAGCTTTTTCACGCTCTATGGACTGCATCTCTCAAAACAGCTCTGGACTGAAAGAACGTAACTACCTAGGATTGTCAGATTGTTCTTCAGTCGACAGCTCCACGGTCTCGAACTTGTCAGAGGGGACTAAGAACAATCTGAACTTTAAGGCTACGGAGTTGAGGCTCGGCCTTCCTGGATCCCAATCACCAGAACGGGAACCTGATCTTTGCTTGTTGAACTCGGGGAAACTTGACGAGAAGCCACTGTTTCCTTTGCTTCCTTCAAAAGATGGAATCTGCTCCTCATCACAGAAGAATGGCAACAAAAGGGGTTTTGCTGACACTATGGATGGATTCTCAGAGGTGAAAAGTAATGCATATACTGAAGGAAATTGGATGTTTCATGCAGCTGGCTCTGATTCTGAATCTCCAGAATCTGTTGGACAAGGGAAGTTTCCTGTGAATTCAATCAATGTAATGCTATCATCCAGGCCTTCTGGGTGTCAGCCAACCATAACAAAAGAAGCACGCACTAAACAAGAACAATCTAATGCTACAAACGGAGGCAACCATAACCCTTTGGGTGCTTCTAACAATGGAAGTGCCCCAGCTGCTAAGGCACAAGTTGTTGGATGGCCTCCAATTAGATCATTTAGGAAGAATTCTCTAGCCACTACATCAAAGAACAATGATGAAGTGAATGGAAAACCTGGTCCCGGTGGACTTTTTGTCAAGGTCAGCATGGATGGGGCTCCCTACCTGAGGAAGGTAGACCTGAGAACCTACTCTACATATCAAGATCTGTCTTCTGCTCTGGAAAAGATGTTCAGCTGTTTTACCATAGGGCAATACGGGTCCCATGGAGCTCCGGGGAGGGAAAGACTGAGTGAGAGTAAGCTGAGAGATCTTCTTCACGGATCAGAATATGTTCTTACATATGAGGACAAGGATGGTGACTGGATGCTTGTTGGGGATGTCCCATGGGAGATGTTTATTGACACATGCAAGAGGTTGAAGATAATGAAGGGCTCCGATGCCATTGGTTTAGCTCCTAGGGCCATGGAGAAATCCAAGAACCGGAACTAG

>MdIAA8

ATGTCTATATCTTTGGAGCATGATTATATAGGCTTATCTCCTTCAATGGAAACCTCTACCAAGTCTGCTGCTTTGAACCTCAAAGCCACTGAGCTGAGGCTGGGTTTGCCTGGCTCTCAGTCTCCAGAGAGAGACGGCTGCGGCGGCGGCGGTAGTGGGGTGGAGGAGAAGGCTACTGGGTTTTCAGTCTGTGGGGTTAAGGGGTTGGTGTCTGGGGCCAAGAGGGGCTTCTCAGACGCCATTGATGGAGCTTCTGGGAAGTGGGTTTTCTCTGGGAGTGGTGGATCTGAGGTGGAGTTAGGCAAAGGTGGGAACTTGCTCTCTCCCAGAGGTGTGAATGCTGGGAAAGCTCTTGCTGCTGGGTGTGAACCCAGCAATCAACCAACAGGTTTAGCTGGCTCTGCTGTGAAAGATGGTGTCCAACAATCTCCAAAGCCATTGCATGAGAAGAAATCCCAAGGGTCTGCTGGGTCTACTGCCCCTGCTGCAAAGGCACAGGTTGTAGGATGGCCACCAATTCGTTCTTTTAGGAAGAATTCAATGGCTTCCGTTCCTTCAAAAAATGGTGATGATGCAGAAGGCAAGATGGGAGCAGGGTGTCTGTATGTTAAGGTCAGCATGGATGGTGCACCGTACCTGAGGAAAGTTGATCTCAAAACCTATGGCAGCTATCTGGATTTATCTCTAGCTCTGGAAAAGATGTTCAGCTGCTTCACAATTGGTCAATGTGGTTCACATGGAGCTTCAAGGGATGGATTGAGCGAGAGTCGATTGATGGACCTCCTACATGGTGCTGAATATGTCCTCACCTATGAAGACAAGGATGGTGATTGGATGCTAGTTGGTGATGTTCCCTGGGAAATGTTCACCGACTCGTGTAAGAGGATGAGGATCATGAAGAGTTCAGAGGCTATCGGTCTAGCCCCAAGGGCCATGCAGAAGTGCAAAAATAGTAATTAG

>MdIAA9

ATGTCTCCGCCACTGCTTGGTGTTGATGAGGAGGAGGGTCAGAGCGATGTTACATTATTGGCTTCTTCGGGTTCTATGGAGAGTGTATGCCAGAACAGTTTGGAACTGAAGGAGCGAAATTACATGGGATTGTCTGATTGCTCTTCGGTAGACAGTTCGAAGGTCTCTGCTGTCTCTGATGGAAGTAAAAGTAGTCTGCATTTGAAGGCTACAGAACTTAGACTTGGGCTTCCTGGATCCCAGTCTCCTGAGAGAGACTCAGAGGCGAGGGTAATCTCCACTCAACTTGATGAGAAGCCCTTATTCCCGTTGCATCCTTTAAAGGATGGCCACTATTCTTCATTGCAGAAAACAGTTGTTTCGGGAAACAAGAGAGGTTTCTCCGATGCTATGGATGAGTTCTCAGAGGGGAAATATGCTAATTCAGAGGTAAACCTGTTACTGTCACCCAGACCTTCTCCAAACTTTGGACTGAAATCTGGTTCTGCACTTGAGAACCCTGGGACTCAACCACCCAAAACAAAAGAGGTGGCACCAGCAAAGGTAGTACAAGAGAGGCCTCATGCTGTCAACAAAAGCAGACCGAATCATAATGAAAATAGTACTAGCGGTGCACCTGCTTCCAAGGCGCAGGTTGTGGGTTGGCCACCTATAAGATCATTTAGGAAGAACTCATTGGCCACTACATCAAAAAACACTGAGGAAGTAGATGGAAAATCGGGCCCTGGTGCTCTGTTTGTCAAGGTCAGCTTGGATGGTGCACCTTATTTGAGGAAAGTAGATTTGAAAAATTACTCTGCATACCAGGAACTTTCTTCTGCTCTTGAAAAGATGTTCCGCTGTTTTACCCTAGGTCAATATGGATCTCATGGAGCTCCAGGCAGAGAGATTAGCGAAAGCAAGCTGAAAGATCTGCTTCATGGCTCAGAATATGTTCTAACTTACGAGGACAAAGATGGTGACTGGATGCTCGTGGGCGATGTTCCTTGGGACATGTTCATTGATACCTGCAAAAGGATGAGGATTATGAAGAGCTCAGATGCCATCGGCCTAGCACCAAGGGCCATGGAAAAGTGCAGGAACAGGAACTAG

>MdIAA10

ATGGCAATGTTTGCGCAGGATCTTGAAGCCACAGAGCTAAGATTAGGGTTACCGGGCACCAAGGATTCCGAGCAGAACACATATTCTACTACTTTGATTAGCAAGAGCAACAAAAGACCACTGCAACCCGACATGAACGACGAAGACAATGATTCTTCATCCGCCAAAAGATCCGACCAGCAAATTTCTCAGCCTCCACCTTCCAAGGCACAAGTAGTAGGGTGGCCGCCGGTGAGATCATACCGGAAAACCTGCCTCCAAGCGAAGAAAACCGAGGCGGAGGCAGCCGGGATTTATGTCAAGATAAGCATGGATGGAGCTCCTTATCTAAGAAAGATAGATTTGAAGGTGTACAGAGGGTATACTGAACTGCTTAAAGCGTTGGAAGACATGTTCAAGTTCAAAGTTGGTGATTATTGTGAGAAGGATTTAGGGTACAATAACAGATCAGAATTTGTACCTACTTATGAAGACAGAGATGGAGATTGGATGCTGCTTGGAGATGTTCCGTGGGAGATGTTCATCGCCTCGTGCAAGAGGTTGAGAATCATGAAGGGTTCGGAAGCGAAAGGCTTAGGCTGCTGCCCGCTCTGA

>MdIAA14

ATGGATAATTTGTATAGTATGATTTGTGAGAATAATGGAGTTGTAAGGGCAAATAAGAGAGGGTATGTTGCAGCTGAAGACAAAAAGCTGGAGCTGAGGCTGGGTCCTCCTGGAGGAGAAGATCATCAGTCACTTCTTTCCCTTGGTTGCTGCAACAAGAACAATATTTCTCATGAAGCCAAAAGAGTCTGTCATGAAGAGAAAAAAGAAGAGAGAAAGTGGCTGGCAAACAGCTCATCAGATCCTCCAGCAAGTGCTTTTGAGCTTACAAATTCAACAGCAGCTCGTGACTCTGATCAGAAAAGTAAAAGCAGAATTGCACAAGCTCCAGTTGTCGGATGGCCTCCGATCCGTTCATCCAGGAAAAATCTTGCAATCAGATCATCACCATCAAGCTTCGCAAAGCCGGCAGCTGATTCGGAGTCACCAAATGAAACTTTGAAGGAGGGAAACGGAAAATCTGATAGTACTACTTATTCCAAGGACCACATGTTTGTAAAGATCAACATGGAAGGAGTTCCCATTGGAAGAAAAATTAACCTCAAAGCCTATGATAGTTATGAGAAACTCTCTTTTGCCATAGATGAACTCTTCCAAGGTCTTCTTGCAGCTCAAAGAACTTGTTGTGGTGTGGAGAAAGAAGACAAGAAGGGAGAGACCAAATCAATAACTGAATCATTACATGGCAATGGGAAATATACTCTACTTTATGAGGATAATGAAGGAGACAGGATGCTTGTTGGTGATGTCCCATGGAACATGTTTGTATCGACGGCAAAGAGGCTTCAGGTATTGAAGAGCTCACAGCTTTCCACTCTACAGCTTAGTAGCAATCAGCGTGAAAAGACACCACTTGATTCTACAATGGAAGTTGGAATTGGAAGATGA

>MdIAA15

ATGGTTAAGTTGTATAATATGATTTGTGAGAATAATGAAGAAGTGGTTGTGAGAGCAAATAAGAGAGGGTATGTTGCAGCTGAAGACAAAAAGCTGGAGCTGAGGCTGGGTCCTCCTGGAGGAGAAGATCGTCAGTCACTTCTCTCCCTTGGTTGCTGCAACAACAATATTCCTCATGAAGCCAAAAGAGCCTGTCATGAAGAGAAAAAAGAAGAGAGAAAGTGGTTGGCAAACAGTTCATCATCAAATCCTGCAGCTAGTGCTTCTGAACTTACGAATTCAGCTGCAGCTCGTGACTCTGATCAGAAAAGTAAAAGCAGAATTGTACATGCTCCAGTTGTCGGGTGGCCTCCGATCCGTTCATCCAGGAAAAATCTTGCAAACAGATCATCATCATCAAGCTTTGCAAACCCGGCAGCTGATTCGGAGTCACCAAATGAAACTTCGAAGGAGGGAAATGGAAAATCTGATGGTACTAATTATTCCAAGCACCACATGTTTGTAAAGATCAACATGGAAGGAGTTCCCATCGGAAGAAAAATTAACCTCAAAGCCTATGATAGTTATGAGAAACTCTCTTTTGCCATATATGAACTCTTTCAAGGTCTTCTTGCAGCTCAAAGAGTTTGTTGTGGCGTGGAGAAAGAAGACAAGAAGGGAGAGACCAAATCAAAAACTGAATCATTACATGGCAGTGGGGAATATACTCTACTCTATGAGGATAATGAAGGGGACAGGATGCTTGTTGGTGATGTCCCATGGAACATGTTTGTATCCACGGCAAAGAGGCTTCGGGTATTGAAGAGCTCACAGCTTTCCACTCTACAACTTAGTAGCAGTCCGCATGAAAAGACACCACTTGATTCTACAGTGAAAGTTGGAATCTGA

>MdIAA16

ATGTTACCGGAAAATAAGCAGCAGCCGCCGGACGGACTGAACTACGATGAGACGAAGCTGACCTTAGGGTTGCCTGGTTCGGGCTCGAAACGTGGATTCTCAGAGACCGTTGATATTAGTCTTGGGAGCTCGAGCTCATCGTCGCCAAGAGCAGGGGTGGAATGTTGTGATCAATATAGTGTTGTTGATGGTGGGGACAAAACTCATAAGGCTCCAGCTAAGGCACAAGTGGTTGGATGGCCACCAGTGAGAGTTTCAAGGAAGAACTTGATGAACAGCTGCAAATATGTGAAAGTGGCAGTTGATGGAGCTCCATATCTGCGCAAGGTTGATCTTGAGATGTACAACAGCTATCAGCAGCTTTTGGGTGCTCTTGAGGACATGTTTTCCTTCTTAAAAATCCGTAATTACTTAAATGAGAGCAAGCTTATGGACCCTGCAAATGGGGTGGAATATGTACCAACTTATGAAGACAGAGATGGCGATTGGATGCTTGTTGGAGATGTCCCATGGAAAATGTTTGTGGAAACATGCAAGCGGCTCCGGTTGATGAAAAGCTCAGAGGCGATCGGATTAGCTCCGAGGACGCCTCCGCAATGCACAAGCACACACTGA

>MdIAA20

ATGGCAATGTTTGCGCAGGATCTTGAAGCCACGGAGCTAAGATTAGGGTTACCAGGCACCAATGATTCCGAGCAGAACACCTCTTCTCCTACTTTGGTTAGCAAGAGTAACAAAAGACCACTGCAATCCGACATGAACGAAGACAATGATTCTTCACCCGCCAACAGATCCGACGAGCAAATTTCTCAGCCTCCACCTACCAAGACACAAGTAGTAGGGTGGCCGCCGGTGAGATCATACTGGAAAAACTGCCTCCAATCGAAGAAAACCGAGGCGGAGGCAGCCGGCATCTACGTGAAGATAAGCATGGATGGAGCTCCTTATCTAAGAAAGATTGATTTGAAGGTGTACAGAGGATATCCTGAGCTGCTTAAAGCATTGGAAGACATGTTCAAGTTCAAGGTTGGTGATTACTGTGAGAAGAAATTAGGGTATAATAACCGATCAGAATTTGTGCCTACTTATGAAGACAAAGATGGAGATTGGATGCTACTTGGAGATGTTCCATGGGAGATGTTCATCGTTTCGTGCAAGAGGTTGAGAATCATGAAAGGTTCGGAAGCGAAAGGGTTAGGCTGCTGCACGCCGTGA

>MdIAA21

ATGGGGTTTGAAGAGACAGAGCTGAGGCTAGGGCTGCCGGGTGGTGGTGGTGGCGGTGGCCGTGACGGAGATCAGGTGGTGGTGATGAGGAAGAGAGGGTTTTCGGAAACTGAGAGTAAGATTAGTACTGATACTAGTACTTGTGTGGATTTGAAGCTTAATCTTTCTAATAGTTCTAAGGAGGCAAATAGTACTGGTGGGAAAGATGGCAGTGCTGAAAAGTCCAAGACCAACAAGGAGAAGAACAACAACCTTGATTTTCGGGCTTCCGATCCAGCAAAGCCTCCTGCCGCCAAGGCACAAGTTGTGGGTTGGCCACCGGTGCGATCTTTTCGAAAGAACATGTTCACGGCGGTGCAAAAGAGCACGAATGATGGAGAAAGTGAGCAGATGAACAAGGGCAGCAATAATAATGCAGTTTTGGTGAAGGTTAGCATGGATGGTGCACCATACCTTCGCAAGGTCGACTTGAAGATGTACAAGAGTTACCCTGAGCTCTCTGATGCCCTAGCCAAAATGTTCAGCTCCTTCACCATTGGAAATTGTGGATCCCAAGGAATGAAGGACTTCATGAATGAGAGAAAGCTGATGGATGTTCTCAACGGTTCTGATTACATTCCAACATACGAAGACAAGGATGGTGATTGGATGCTGGTTGGCGATGTGCCATGGGAGATGTTTGTTGAATCATGCAAGAGATTGCGCATAATGAAGAGCAAGGAGGCTGTTGGACTAGCACCAAGGGCCATGGAGAAATGCAAGAACAGGAGCTGA

>MdIAA24

ATGTCTAGGCCACTGGAGCTTGATTACATAGGCCTAACAGAGACTTCATCTTCTCCAATGGAAAGAAGCTCTGAGAAGATTTCATCTTCCACTTCCTCTACCTTCTCTTCCACAACAGCTGAGGAAAAGAGGACCTCCTCCTCTGCTATCAACCTCAGGGAGACTGAGCTCAGGCTCGGCCTTCCTGGTTCCCACTTCCCTGACAGAAAAAAACTGTCAGGGGTCTCAATTTTTGGGAAAGATTTGGAGGACAACAACAAGCCAAATGGGTGTTCTCCAAATCCTCCAAAGAACCCTGTGTCAGGGGCAAAAAGGGGATTCTCCGACGCCATTGATGGGTGTGAGAAATGGGTTTTCTCCATGGGTAATGGATCTGAGGTTGATGTGGGTAAAGCAGCAGTGTTGGGTTCTCCATTAGCCAAGAAAGAGGTTTCTTCTGTTCCTCTGCCACCAAAGCCATCAGCTCAGTTGGAGAAGAAGAAGCCTCAGGCCTCTGAGCATGCTGCTGCTCCTCCTGCTGCCAAGGCACAGGTGGTGGGATGGCCCCCAATTCGATCGTTTCGGAAGAACTCCATGGCCTCTAATTTGGCAAAGAACAACGACGATGCTGAGGGCAAACAAGGGTCCGGGTGCCTGTATGTGAAGGTTGGCATGGATGGTGCTCCGTACCTACGAAAGGTTGACCTTAAAACGTACAACAACTACACTGAACTTTCCATGGCTCTGGAGAAGATGTTTAGCTGCTTCACCATTGGGCAGAGCAGTTGTAATGGACTTCCGGAGCGAGATGGTCTGAGCGCGAGTCGTCTGATGGATCTTCTCAACGGTTCTGAATTTGTTCTGACTTATGAGGACAGGGATGATGACTGGATGCTTGTTGGTGATGTTCCTTGGCAGATGTTCACCGAGACCTGTAGGAGGCTGAGGATCATGAAGGGTTCCGAAGCAATCGGTCTAGCTCCAAGGGCTGTGGAGAAATGCAAGAACCGGAACTAA

>MdIAA26

ATGACGAGCATGCTTGGAGTTGAGTGTGATTTGAACCTCAGAGAGACTGAGCTGTGCCTAGGGCTGCCTGGTGGTGGTACTTCTACTGTGGCTGAGCCGGAAACTAAGGCTACCGGAAAGAGAGGCTTCTCTGAGACCGTTGATCTCAAGCTCAAGCTTCAGTCCAAGGATGATCTGAATGACAATGTAAAAAATATTGCTTCCAGGGAGAAGAACGACCTCCTTTCTTGCACAAAAGATCCAGCAAAGCCTCCAGCCAAGGCACAAGTTGTGGGTTGGCCACCAGTTCGATCATACCGAAAGAACATAATGGCGCAGAAGAACACCAGCGAGGAAAATACTGAGAAGGCAACCGTCGGTGGCGGAGGCTGCAGCGCTGCGTTTGTGAAGGTTTGCATGGATGGCGCTCCATATCTTCGTAAGGTGGATTTGAAGATGTACAAGAGTTACCAAGAGCTCTCTAATGCCTTAGCCAAGATGTTCAGTTCCTTCACCACCGGTTACTATGGGGCCCAAGGAATGATAGACTTCATGAACGAGAGCAAGTTGATGGATCTTCTTAACAGTTCCGAGTATGTGCCAACCTATGAAGATAAGGACGGTGATTGGATGCTCGTGGGTGACGTTCCATGGGGGATGTTCGTTGATTCATGCAAGCGCTTGCGAATAATGAAAGGATCAGAAGCAATTGGACTTGCCCCAAAAGCTATGGAGAAATGCAAAGGCAGAAGCTGA

>MdIAA27

ATGGCCACTGAGATGGAGGGATGTACAAGGAAGGAGAAGGAGGCATGTCCACAACTGCTAGATTTGATTCCAAGAGAGAGAGACTGGCTTGTGAGCAGATATATTGAGAGAAGCAGCGGCCATGGAAGCTCCTCCTCAGAGGAGAAGAAGCTTGAGCTTAGGCTTGGCCCTCCAGGTCAAGACTGGTCAATGAAAAGAGAAAGAGATGAGAAGTCTCAGTCCCTTGTCTCTCTTGGATACTTTTCTTCCCCCATGTCCTCCAACCAAACCCAGAAATTTATTTCTTCTTACCAGCAGCAGGCAAAAGTTGCGTCTTTTCTTCACCTACCTGTCACCCCCAACAAAGCGTCACAGCCCTGGTGCACTAAAGCAGTAGACTTGCAGAATGCAGAAAATAAAGGGTTTTCACCAGCCCCTGCAAATACAGCTGTGCTCCCCAACACCTCTCAGAAAAGGAAGAATCTTGCAAGCAACAAAGGCTCATCAAAACCAGCAGTCGAATCCGAAAATGTTGTCCAAATCAAAGATCCTAATGGTAAATCAGTTGAAACCAGCGGAAAGGGTCTGTTTGTGAAAATTAACATGGATGGAGTCCCCATTGGAAGAAAAGTGGACCTCGGTGCTTATGACAGCTACCAAAAGCTCTCCTCCGCTGTCGATGAACTCTTCCGTGGCCTTCTCGCCGCTCAAAGAGAATCCTGTAATGGTGGAACCAAGAACAAGCAAGAGGAAGAGAAAGAAATCACAGGCTTATTGGATGGAAGTGGAGAATACACCCTTGTTTATGAAGATAATGAAGGAGACAGGATGCTTGTTGGGGATGTCCCATGGCACATGTTTGTGTCTACTGTGAAGAGGCTGCGTGTGCTGAAGAGCTCTGAACTTTCTGCACTTCGTCTTCGTAGCAGCAAAGATAAGATGGCACTTTGA

>MdIAA28

ATGGAAGACCAGCTAAATTTCAAGGCAACTGAGCTAAGATTAGGGTTGCCTGGAAGCTCTGAAGAGCCTGAGAACAAAAAAGCAGCACCGTCTCCTCCTATGGCTAAGAATAACAAGAGGGCTTCGCCGGATTCAGCGGCTGAGGAGTGCAGTACAAGTTCTGATCCCATAGATGTCCCTCCCACCAAGACACAGGTAGTAGGGTGGCCTCCAATCAGATCTTACAGGAAAAACAGTTTGCAACTGCGAGAGGCGTACGTGAAAGTAAGCGTCGACGGAGCTCTTATCTGAGGAAGATTGATCTCAAGGTTTACAATAGCTACCCCGAACTCATCAAGGCCTTAGAGAAGATGTTCAACCTAGCCAATATTAATGGATCCGATTTTGCTCCGACATATGAAGACAAAGACGGCGACTGGATGCTTGTTGGAGATGTGCCATGGAATATGTTTGTTTCTTCCTGCAAAAGGCTGAGAATCATGAAGGGATCTGAAGCCAGAGGATTGAGCACTTGTTTCTGA

>MdIAA30

ATGGAAAGCAAGGCACATGAGAAAGATCTCAACCTCAAGGCAACAGAGCTGACACTACGGTTGCCAGGGAGAGATGAAATTATTGAGCTTCCTAATCAAAACAAGAAGAGAGGACTGCCAGCTGACTCAAAAAATGAAGAAGGAAGCACTGATGCTAAAAACGCTCAAAAGGAAACCCCTCCTGCAAAGGCACAAATAGTGGGGTGGCCACCAATCAGATCTTATAGGAAGAACAGCCTCCAAGCAAATAAAAATGCAGAGGGTGAGACTGCTACCGGGATTTATGTGAAAGTGAGCATGGATGGAGCACCTTACCTCAGAAAGATTGATCTTAAGGTTTACAAATGCTACCCAGAACTCCTCAAGGCCTTGGAAGTCATGTTTAAGTTTACTATAGGTCAATACTCAGAGAGGGAGGGCTACAAAGGATCAGAATATGCACCTACTTATAAGGACAAAGATGGTGATTGGATGCTGGTTGGAGATGTTCCATGGGATATGTTCATGTCATCCTGCAAGAAGTTGAGAATCATGAAAGGATCAGAGGCCAGAGGTTTGGGGTGTGGCGTATGA

>MdIAA31

ATGCAAACACAGACACAAGCGTCCCACGCAGTTATATTATTGTGTGGGGGGTCTCCCTCTCTCCTAGCTCAAAACCAACACAACAACGCAACAAGACAGAAGAAAGAAGAAGAAGCAGCGAAAGCAGCTCTACGCATCACCACCAATATAAACGCAAGCTTACAGATCCTCACAGGCTTTTTATTAAAGCACTTTCTTCAAAATCTCTATTTTCTTGTTAGCCGGAAGATGATGAGCATGCTTGGAGTTGAGCGTGATCTGAACCTCCGAGAGACTGAGCTGTGCCTAGGGCTGCCCGGTGGCGGTACTACTACTGTGGCCGAGCCGGAGACTGCCAAGACTACTGGAAAGAGAGGGTTCTCGGAGACCATTGATCTCAAGCTCAACCTTCAGTCCAAGGAAGATCTGAATGACAATGTAAAAAATATTGCTTCCAAGGACAAAAACAACTTTCTTTCTTGCACAAAAGATCCATCAAAGCCTCCAGCCAAGGCACAAGTTGTGGGTTGGCCACCAGTTCGATCATACCGAAAGAACATAATGGCGCAAAAGAATACCAGCGAGGAAAAAACTAAGAAGGCAAGCGGCGGCGGTGGCTGCACCGCCGCGTTTGTGAAGGTTTGCATGGATGGCGCTCCATATCTCCGTAAGGTAGATTTGAAGATGTACAAGAGCTACCAAGAGCTCTCTAATGCCTTAGCCAAGATGTTCAGTTCCTTCACCACCGGTTACTATGGGAGCCAAGGAATGATAGACTTCATGAATGAGAGCAAGTTGATGGATCTTCTTAACAGTTCTGAGTATGTGCCGACCTATGAAGATAAGGATGGTGATTGGATGCTCGTGGGTGATGTTCCATGGGGGATGTTGGTTGATTCATGCAAGCGCTTGCGAATAATGAAAGGATCAGAAGCAATTGGACTTGCCCCAAAAGCTATGGAGAAATGCAAGGGCAGAAGCTGA

>MdIAA33

ATGTCACCGCCACTGCTTGGTGTTGGTGAGGAGGAGGGTCAGAGCGATGTTACATTTTTGTCTTCTTCGGGTTCTATGGAGAGTGTATGCCAAAACAGTTCGGAATTGAAGGAGCAAAATTACATGGGATTATCTGATTGTTCTTCGGTAGGCAGTTCAAATGTCTCTACAGCCTCTGATGGAAGTAATAGTAGTCTGAATTTGAAGGCCACAGAACTTAGACTCGGGCTTCCTGGATCCCAGTCTCCCAAGAGAGACTCAGATTCGAGGGTAATCACCACTCAACTTGATGAGAAGCCCCTATTCCCGCTGCATCCTTTAAAGGATGGCCACTATTCTTCATCGCAGAAAATAGTTGTTTCGGGAAACAAGAGAGGTTTCTCCGATGCTATGGATGAGTTCTCAGAGGGGAAATATGCTAATTCAGAGGTAAACCTGTTGCTGTCACCCAGACCTTCTCCAAACTTGGGACTGAAATCTGGTTCTACACTTGAGAACCTTGGGACTCAACCACCCAAAACAAAAGAGGTGGCACCAGCAAAGGTAGTACAAGAGAGGCCTCATGCTGTCAATGATACCAGATCGAATCATACCGGAAATAGTACTAGGAGTGCACCTGCTTCCAAGGCGCAGGTTGTGGGTTGGCCACCTATAAGATCATTTAGGAAGAACTCATTGGCCACTACATCAAAAAACACTGAGGAAGTAGATGGAAAATCGAACCCTGGTGCTCTGTTTGTCAAGGTCAGCTTGGATGGTGCACCTTATTTGAGGAAAGTAGATTTGAAAAATTACTCTGCATACCAGGAACTTTCTTCTGCTCTTGAAAAGATGTTCAGCTGTTTTACCATAGGTCAATATGGATCTCATGGAGCTCCAGGCAGAGAGATTAGCGAAAGCAAGCTGAAAGATCTGCTTCATGGCTCAGAATATGTTCTAACTTACGAGGACAAAGATGGTGACTGGATGCTTGTGGGCGATGTTCCTTGGGACATGTTTACTGATACCTGCAAAAGGTTGAGGATTATGAAGAGCTCAGATGCCATCGGCCTAGCTCCTAGGGCCATGGAGAAGTGCAGGAACAGGAACTAG
