# Supplementary material for: Overexpression of MdIAA9 confers high tolerance to osmotic stress in transgenic tobacco
Source: PeerJ. 2019 Oct 31;7:e7935. doi: 10.7717/peerj.7935 (PMC6825743; doi:10.7717/peerj.7935)
Supplement: Supplemental Information 2 [file peerj-07-7935-s002.docx]

**ABA**

|  | IAA4 | IAA5 | IAA8 | IAA9 | IAA10 | IAA14 | IAA15 | IAA16 | IAA20 | IAA21 | IAA24 | IAA26 | IAA27 | IAA28 | IAA30 | IAA31 | IAA33 |
| --- | --- | --- | --- | --- | --- | --- | --- | --- | --- | --- | --- | --- | --- | --- | --- | --- | --- |
| 0h | 1.00 | 1.00 | 1.00 | 1.00 | 1.01 | 1.01 | 1.02 | 1.00 | 1.00 | 1.00 | 1.00 | 1.00 | 1.00 | 1.00 | 1.01 | 1.00 | 1.02 |
| 2h | 1.23 | 1.07 | 0.70 | 1.26 | 0.49 | 1.00 | 0.50 | 2.49 | 0.37 | 1.04 | 1.31 | 0.97 | 1.03 | 1.01 | 0.45 | 1.07 | 1.39 |
| 4h | 0.88 | 0.95 | 0.60 | 1.14 | 0.19 | 0.31 | 0.34 | 1.09 | 0.13 | 0.31 | 0.69 | 0.63 | 0.47 | 0.89 | 0.12 | 0.51 | 0.77 |
| 8h | 0.69 | 1.64 | 1.47 | 1.34 | 0.26 | 0.34 | 1.47 | 0.57 | 0.18 | 0.26 | 0.42 | 0.58 | 1.09 | 1.34 | 0.07 | 0.47 | 0.66 |
| 12h | 0.72 | 1.66 | 2.42 | 1.18 | 0.64 | 0.36 | 2.12 | 0.31 | 0.46 | 0.74 | 0.58 | 0.78 | 1.45 | 1.53 | 0.08 | 0.76 | 1.05 |
| 24h | 1.84 | 1.09 | 1.13 | 1.42 | 0.35 | 0.44 | 0.47 | 1.34 | 0.24 | 0.38 | 1.38 | 0.63 | 0.52 | 0.70 | 0.23 | 0.55 | 1.80 |
|  |  |  |  |  |  |  |  |  |  |  |  |  |  |  |  |  |  |
|  | IAA4 | IAA5 | IAA8 | IAA9 | IAA10 | IAA14 | IAA15 | IAA16 | IAA20 | IAA21 | IAA24 | IAA26 | IAA27 | IAA28 | IAA30 | IAA31 | IAA33 |
| 0h | 0.06 | 0.05 | 0.10 | 0.06 | 0.13 | 0.14 | 0.22 | 0.06 | 0.02 | 0.08 | 0.06 | 0.01 | 0.02 | 0.06 | 0.15 | 0.03 | 0.06 |
| 2h | 0.05 | 0.03 | 0.08 | 0.04 | 0.03 | 0.11 | 0.02 | 0.08 | 0.03 | 0.09 | 0.07 | 0.10 | 0.09 | 0.06 | 0.03 | 0.06 | 0.03 |
| 4h | 0.10 | 0.12 | 0.09 | 0.08 | 0.01 | 0.02 | 0.05 | 0.08 | 0.01 | 0.02 | 0.04 | 0.06 | 0.04 | 0.08 | 0.00 | 0.03 | 0.09 |
| 8h | 0.02 | 0.09 | 0.12 | 0.06 | 0.01 | 0.05 | 0.08 | 0.02 | 0.02 | 0.03 | 0.05 | 0.03 | 0.11 | 0.03 | 0.02 | 0.04 | 0.09 |
| 12h | 0.05 | 0.21 | 0.31 | 0.18 | 0.09 | 0.07 | 0.06 | 0.04 | 0.05 | 0.06 | 0.12 | 0.03 | 0.05 | 0.17 | 0.01 | 0.14 | 0.12 |
| 24h | 0.24 | 0.14 | 0.07 | 0.05 | 0.03 | 0.06 | 0.02 | 0.12 | 0.03 | 0.05 | 0.12 | 0.11 | 0.05 | 0.01 | 0.01 | 0.01 | 0.22 |

**COLD**

|  | IAA4 | IAA5 | IAA8 | IAA9 | IAA10 | IAA14 | IAA15 | IAA16 | IAA20 | IAA21 | IAA24 | IAA26 | IAA27 | IAA28 | IAA30 | IAA31 | IAA33 |
| --- | --- | --- | --- | --- | --- | --- | --- | --- | --- | --- | --- | --- | --- | --- | --- | --- | --- |
| 0h | 1.01 | 1.01 | 1.01 | 1.00 | 1.00 | 1.00 | 1.00 | 1.01 | 1.01 | 1.01 | 1.00 | 1.00 | 0.98 | 1.00 | 1.01 | 1.00 | 1.01 |
| 2h | 1.45 | 1.10 | 0.95 | 1.10 | 1.08 | 0.83 | 1.33 | 0.65 | 2.03 | 0.88 | 1.26 | 0.22 | 2.02 | 1.27 | 0.86 | 0.75 | 1.21 |
| 4h | 1.51 | 1.13 | 0.81 | 1.10 | 2.16 | 1.17 | 1.88 | 0.94 | 4.13 | 4.80 | 0.91 | 1.45 | 3.33 | 1.89 | 1.41 | 1.17 | 0.82 |
| 8h | 1.37 | 0.86 | 0.87 | 0.83 | 1.87 | 0.59 | 1.96 | 0.65 | 1.46 | 0.11 | 1.09 | 0.42 | 0.33 | 0.90 | 1.43 | 0.74 | 1.09 |
| 12h | 1.34 | 0.61 | 0.63 | 0.66 | 1.35 | 0.38 | 0.86 | 0.71 | 2.20 | 0.15 | 1.22 | 0.38 | 0.34 | 0.96 | 1.30 | 0.41 | 1.04 |
| 24h | 1.86 | 0.59 | 0.62 | 0.60 | 1.05 | 0.18 | 0.48 | 0.69 | 3.26 | 0.43 | 1.14 | 1.66 | 0.95 | 1.70 | 0.92 | 0.27 | 1.06 |
|  |  |  |  |  |  |  |  |  |  |  |  |  |  |  |  |  |  |
|  | IAA4 | IAA5 | IAA8 | IAA9 | IAA10 | IAA14 | IAA15 | IAA16 | IAA20 | IAA21 | IAA24 | IAA26 | IAA27 | IAA28 | IAA30 | IAA31 | IAA33 |
| 0h | 0.14 | 0.14 | 0.17 | 0.03 | 0.03 | 0.06 | 0.08 | 0.20 | 0.18 | 0.21 | 0.10 | 0.03 | 0.20 | 0.07 | 0.16 | 0.09 | 0.17 |
| 2h | 0.16 | 0.07 | 0.09 | 0.03 | 0.04 | 0.02 | 0.18 | 0.04 | 0.37 | 0.11 | 0.06 | 0.02 | 0.08 | 0.09 | 0.12 | 0.02 | 0.12 |
| 4h | 0.15 | 0.11 | 0.11 | 0.10 | 0.26 | 0.46 | 0.01 | 0.02 | 0.41 | 0.47 | 0.16 | 0.08 | 0.19 | 0.12 | 0.20 | 0.29 | 0.02 |
| 8h | 0.09 | 0.02 | 0.27 | 0.05 | 0.06 | 0.05 | 0.12 | 0.04 | 0.27 | 0.05 | 0.09 | 0.08 | 0.13 | 0.14 | 0.06 | 0.15 | 0.13 |
| 12h | 0.05 | 0.03 | 0.06 | 0.05 | 0.15 | 0.04 | 0.07 | 0.17 | 0.32 | 0.07 | 0.08 | 0.11 | 0.16 | 0.13 | 0.20 | 0.11 | 0.05 |
| 24h | 0.07 | 0.03 | 0.05 | 0.03 | 0.08 | 0.02 | 0.03 | 0.06 | 0.62 | 0.09 | 0.06 | 0.15 | 0.28 | 0.08 | 0.06 | 0.02 | 0.08 |

**DROUGHT**

|  | IAA4 | IAA5 | IAA8 | IAA9 | IAA10 | IAA14 | IAA15 | IAA16 | IAA20 | IAA21 | IAA24 | IAA26 | IAA27 | IAA28 | IAA30 | IAA31 | IAA33 |
| --- | --- | --- | --- | --- | --- | --- | --- | --- | --- | --- | --- | --- | --- | --- | --- | --- | --- |
| 0d | 1.00 | 1.01 | 1.01 | 1.02 | 1.01 | 1.00 | 1.01 | 1.01 | 1.00 | 1.00 | 1.00 | 1.03 | 1.00 | 1.00 | 1.01 | 1.00 | 1.01 |
| 2d | 0.86 | 0.80 | 0.53 | 8.99 | 0.50 | 0.68 | 0.31 | 1.60 | 0.39 | 1.93 | 0.69 | 8.95 | 1.38 | 0.68 | 0.50 | 0.79 | 7.50 |
| 4d | 3.05 | 1.19 | 1.49 | 10.03 | 0.67 | 0.77 | 0.38 | 2.25 | 0.46 | 1.42 | 1.12 | 5.49 | 2.14 | 1.01 | 0.51 | 1.05 | 6.24 |
| 6d | 4.64 | 1.20 | 1.18 | 6.24 | 0.65 | 0.70 | 0.57 | 1.62 | 0.37 | 0.80 | 0.53 | 3.48 | 1.74 | 2.38 | 0.85 | 0.54 | 1.55 |
| 8d | 5.14 | 2.15 | 1.91 | 7.51 | 0.63 | 0.58 | 0.37 | 2.40 | 0.13 | 0.56 | 0.40 | 1.76 | 0.38 | 1.47 | 0.33 | 0.65 | 1.33 |
| 10d | 3.64 | 0.82 | 1.13 | 3.38 | 0.91 | 0.47 | 0.46 | 0.72 | 0.52 | 0.55 | 0.24 | 2.85 | 1.86 | 1.14 | 0.31 | 0.31 | 0.77 |
|  |  |  |  |  |  |  |  |  |  |  |  |  |  |  |  |  |  |
|  | 4 | 5 | 8 | 9 | 10 | 14 | 15 | 16 | 20 | 21 | 24 | 26 | 27 | 28 | 30 | 31 | 33 |
| 0d | 0.05 | 0.17 | 0.20 | 0.08 | 0.12 | 0.11 | 0.18 | 0.15 | 0.12 | 0.09 | 0.05 | 0.29 | 0.15 | 0.09 | 0.14 | 0.03 | 0.17 |
| 2d | 0.06 | 0.15 | 0.02 | 0.31 | 0.04 | 0.02 | 0.10 | 0.02 | 0.22 | 0.37 | 0.09 | 0.60 | 0.17 | 0.05 | 0.16 | 0.10 | 1.24 |
| 4d | 0.36 | 0.17 | 0.27 | 0.45 | 0.06 | 0.01 | 0.03 | 0.35 | 0.06 | 0.11 | 0.06 | 1.08 | 0.17 | 0.11 | 0.09 | 0.23 | 0.59 |
| 6d | 0.16 | 0.15 | 0.08 | 0.36 | 0.04 | 0.05 | 0.12 | 0.17 | 0.04 | 0.04 | 0.02 | 0.43 | 0.18 | 0.10 | 0.17 | 0.03 | 0.24 |
| 8d | 0.55 | 0.11 | 0.13 | 0.41 | 0.07 | 0.08 | 0.02 | 0.03 | 0.05 | 0.09 | 0.04 | 0.24 | 0.08 | 0.22 | 0.11 | 0.02 | 0.14 |
| 10d | 0.28 | 0.08 | 0.02 | 0.76 | 0.08 | 0.02 | 0.07 | 0.07 | 0.36 | 0.08 | 0.02 | 0.40 | 0.22 | 0.06 | 0.04 | 0.00 | 0.07 |

**IAA**

|  | IAA4 | IAA5 | IAA8 | IAA9 | IAA10 | IAA14 | IAA15 | IAA16 | IAA20 | IAA21 | IAA24 | IAA26 | IAA27 | IAA28 | IAA30 | IAA31 | IAA33 |
| --- | --- | --- | --- | --- | --- | --- | --- | --- | --- | --- | --- | --- | --- | --- | --- | --- | --- |
| 0h | 1.00 | 1.00 | 1.00 | 1.00 | 1.01 | 1.01 | 1.00 | 1.00 | 1.00 | 1.02 | 0.99 | 1.02 | 1.03 | 1.06 | 0.92 | 0.92 | 1.00 |
| 2h | 1.17 | 1.12 | 1.20 | 1.63 | 1.02 | 0.67 | 1.03 | 0.62 | 0.76 | 0.86 | 1.08 | 1.72 | 1.02 | 0.88 | 0.56 | 1.48 | 1.30 |
| 4h | 4.87 | 1.42 | 2.41 | 2.15 | 3.93 | 1.44 | 2.04 | 1.35 | 4.87 | 0.82 | 1.02 | 0.97 | 1.19 | 3.66 | 0.80 | 0.97 | 1.43 |
| 8h | 3.66 | 1.22 | 2.64 | 3.11 | 0.70 | 0.34 | 0.68 | 0.46 | 0.90 | 0.35 | 1.11 | 0.67 | 0.88 | 0.80 | 0.14 | 0.59 | 1.22 |
| 12h | 1.60 | 0.90 | 3.44 | 2.69 | 0.57 | 0.09 | 0.72 | 0.17 | 0.78 | 0.23 | 1.60 | 0.38 | 0.63 | 0.57 | 0.13 | 0.35 | 1.44 |
| 24h | 1.62 | 1.68 | 1.63 | 1.51 | 2.00 | 1.69 | 1.71 | 1.05 | 3.59 | 1.36 | 1.04 | 1.19 | 1.13 | 1.79 | 1.27 | 1.26 | 1.13 |
|  |  |  |  |  |  |  |  |  |  |  |  |  |  |  |  |  |  |
|  | 4 | 5 | 8 | 9 | 10 | 14 | 15 | 16 | 20 | 21 | 24 | 26 | 27 | 28 | 30 | 31 | 33 |
| 0h | 0.10 | 0.05 | 0.09 | 0.06 | 0.14 | 0.16 | 0.07 | 0.10 | 0.03 | 0.23 | 0.10 | 0.26 | 0.28 | 0.11 | 0.11 | 0.09 | 0.12 |
| 2h | 0.10 | 0.07 | 0.14 | 0.21 | 0.07 | 0.18 | 0.20 | 0.17 | 0.12 | 0.07 | 0.03 | 0.36 | 0.13 | 0.07 | 0.12 | 0.23 | 0.12 |
| 4h | 0.25 | 0.09 | 0.14 | 0.25 | 0.34 | 0.18 | 0.17 | 0.42 | 0.31 | 0.04 | 0.11 | 0.17 | 0.47 | 0.17 | 0.04 | 0.05 | 0.16 |
| 8h | 0.47 | 0.18 | 0.36 | 0.35 | 0.10 | 0.10 | 0.09 | 0.14 | 0.11 | 0.03 | 0.06 | 0.24 | 0.08 | 0.07 | 0.00 | 0.09 | 0.05 |
| 12h | 0.05 | 0.02 | 0.19 | 0.39 | 0.09 | 0.05 | 0.06 | 0.05 | 0.08 | 0.02 | 0.20 | 0.07 | 0.08 | 0.00 | 0.02 | 0.03 | 0.03 |
| 24h | 0.11 | 0.15 | 0.09 | 0.16 | 0.21 | 0.20 | 0.13 | 0.08 | 0.42 | 0.14 | 0.05 | 0.18 | 0.07 | 0.07 | 0.07 | 0.15 | 0.08 |

**NaCl**

|  | IAA4 | IAA5 | IAA8 | IAA9 | IAA10 | IAA14 | IAA15 | IAA16 | IAA20 | IAA21 | IAA24 | IAA26 | IAA27 | IAA28 | IAA30 | IAA31 | IAA33 |
| --- | --- | --- | --- | --- | --- | --- | --- | --- | --- | --- | --- | --- | --- | --- | --- | --- | --- |
| 0h | 1.01 | 1.01 | 1.02 | 1.00 | 1.00 | 1.00 | 1.00 | 1.01 | 1.00 | 1.01 | 1.00 | 1.00 | 1.00 | 1.01 | 1.02 | 1.00 | 1.01 |
| 2h | 0.74 | 0.74 | 0.69 | 0.91 | 0.26 | 0.72 | 0.48 | 1.38 | 0.20 | 0.94 | 6.82 | 1.42 | 0.85 | 0.96 | 0.42 | 0.96 | 0.70 |
| 4h | 0.62 | 0.67 | 0.58 | 0.93 | 0.14 | 0.47 | 0.52 | 0.94 | 0.18 | 1.27 | 4.49 | 1.47 | 0.83 | 1.26 | 0.25 | 0.77 | 0.97 |
| 8h | 0.89 | 1.75 | 2.47 | 1.75 | 0.34 | 0.86 | 1.97 | 0.74 | 0.32 | 0.72 | 0.64 | 1.43 | 1.32 | 3.22 | 0.56 | 1.02 | 0.39 |
| 12h | 0.73 | 1.27 | 2.85 | 1.13 | 0.60 | 0.45 | 2.02 | 0.27 | 0.48 | 0.75 | 0.85 | 1.15 | 1.25 | 1.34 | 0.26 | 0.83 | 0.61 |
| 24h | 1.01 | 0.78 | 0.71 | 1.20 | 0.25 | 0.58 | 0.55 | 1.63 | 0.24 | 0.79 | 2.58 | 1.44 | 0.89 | 1.25 | 0.39 | 1.01 | 0.99 |
|  |  |  |  |  |  |  |  |  |  |  |  |  |  |  |  |  |  |
|  | 4 | 5 | 8 | 9 | 10 | 14 | 15 | 16 | 20 | 21 | 24 | 26 | 27 | 28 | 30 | 31 | 33 |
| 0h | 0.14 | 0.18 | 0.26 | 0.07 | 0.09 | 0.09 | 0.08 | 0.12 | 0.08 | 0.17 | 0.08 | 0.03 | 0.06 | 0.12 | 0.23 | 0.11 | 0.13 |
| 2h | 0.17 | 0.04 | 0.03 | 0.02 | 0.02 | 0.04 | 0.02 | 0.11 | 0.03 | 0.03 | 0.35 | 0.05 | 0.07 | 0.05 | 0.06 | 0.02 | 0.11 |
| 4h | 0.07 | 0.05 | 0.02 | 0.09 | 0.01 | 0.04 | 0.20 | 0.05 | 0.05 | 0.25 | 0.45 | 0.37 | 0.19 | 0.09 | 0.03 | 0.02 | 0.36 |
| 8h | 0.10 | 0.14 | 0.02 | 0.20 | 0.02 | 0.11 | 0.08 | 0.05 | 0.01 | 0.03 | 0.04 | 0.11 | 0.16 | 0.44 | 0.08 | 0.05 | 0.04 |
| 12h | 0.07 | 0.10 | 0.05 | 0.02 | 0.07 | 0.05 | 0.20 | 0.05 | 0.08 | 0.08 | 0.08 | 0.15 | 0.08 | 0.13 | 0.04 | 0.06 | 0.06 |
| 24h | 0.04 | 0.02 | 0.02 | 0.05 | 0.04 | 0.10 | 0.02 | 0.09 | 0.01 | 0.06 | 0.10 | 0.09 | 0.07 | 0.57 | 0.06 | 0.13 | 0.28 |

**Various tissues**

|  | Flower | Fruit | Leaf | Root | Stem |
| --- | --- | --- | --- | --- | --- |
| MdIAA9 | 11.27 | 11.08 | 9.65 | 10.44 | 10.13 |
| MdIAA33 | 12.76 | 12.57 | 12.13 | 12.68 | 12.78 |
| MdIAA5 | 8.65 | 9.07 | 8.33 | 9.46 | 9.00 |
| MdIAA8 | 8.67 | 9.67 | 8.48 | 8.27 | 8.39 |
| MdIAA24 | 11.97 | 11.68 | 10.39 | 10.77 | 10.72 |
| MdIAA21 | 10.99 | 10.60 | 10.72 | 9.64 | 9.62 |
| MdIAA26 | 8.58 | 8.32 | 8.47 | 8.47 | 8.32 |
| MdIAA31 | 8.90 | 8.87 | 8.84 | 9.19 | 8.96 |
| MdIAA28 | 9.80 | 9.49 | 8.78 | 8.64 | 8.62 |
| MdIAA10 | 10.49 | 10.79 | 10.12 | 10.08 | 9.91 |
| MdIAA20 | 12.88 | 14.83 | 12.01 | 11.82 | 11.00 |
| MdIAA14 | 8.71 | 9.14 | 8.16 | 8.77 | 8.44 |
| MdIAA30 | 9.20 | 9.12 | 8.38 | 9.16 | 8.67 |
| MdIAA16 | 9.50 | 8.47 | 8.91 | 8.08 | 8.28 |
| MdIAA4 | 8.28 | 8.32 | 8.05 | 8.88 | 8.07 |
| MdIAA15 | 8.19 | 8.59 | 8.40 | 8.08 | 8.17 |
| MdIAA27 | 9.37 | 9.69 | 8.82 | 8.81 | 9.26 |

Relative expression analysis of *MdIAA9* in wild-type and transgenic *tobacco* lines

| 1.000693 | 0.037432 |
| --- | --- |
| 1.70921 | 0.176407 |

| root length | |  |  |  | fresh weight | |  |
| --- | --- | --- | --- | --- | --- | --- | --- |
|  | MS | 200 mM Mannitol | |  |  | MS | 200 mM Mannitol |
| WT | 3.53 | 3.83 |  |  | WT | 0.0104 | 0.0056 |
| 9-54 | 3.24 | 4.98 |  |  | 9-54 | 0.0105 | 0.0079 |
| 9-60 | 3.36 | 5.67 |  |  | 9-60 | 0.0113 | 0.0098 |
|  | 0.31 | 0.26 |  |  |  | 0.00203 | 0.00042 |
|  | 0.3 | 0.42 |  |  |  | 0.0014 | 0.0008 |
|  | 0.24 | 0.26 |  |  |  | 0.00157 | 0.001 |
|  |  |  |  |  |  |  |  |
| REL |  |  |  |  | proline content | |  |
|  | MS | 200 mM Mannitol | |  |  | MS | 200 mM Mannitol |
| WT | 21.14 | 59.2 |  |  | WT | 38.81 | 163.67 |
| 9-54 | 24.17 | 44.26 |  |  | 9-54 | 40.8 | 207.94 |
| 9-60 | 24.02 | 40.23 |  |  | 9-60 | 34.2 | 206.6 |
|  | 3.31 | 9.2 |  |  |  | 7.48 | 5.41 |
|  | 2.41 | 4.19 |  |  |  | 3.41 | 16.7 |
|  | 3.25 | 7.13 |  |  |  | 3.64 | 14.13 |
|  | 3.25 | 7.13 |  |  |  |  |  |
|  |  |  |  |  |  |  |  |
| MDA |  |  |  |  | chlorophyll content | |  |
|  | MS | 200 mM Mannitol | |  |  | MS | 200 mM Mannitol |
| WT | 2.07 | 2.83 |  |  | WT | 0.3687272 | 0.2704254 |
| 9-54 | 1.87 | 2.16 |  |  | 9-54 | 0.5545535 | 0.4479653 |
| 9-60 | 2 | 2.33 |  |  | 9-60 | 0.6302501 | 0.4599938 |
|  | 0.26 | 0.27 |  |  |  | 0.0542159 | 0.0185462 |
|  | 0.15 | 0.24 |  |  |  | 0.0860325 | 0.0589826 |
|  | 0.08 | 0.14 |  |  |  | 0.0531886 | 0.0555207 |
